# Dysregulation of Notch-FGF signaling axis in germ cells results in cystic dilation of the rete testis in mice

**DOI:** 10.1007/s12079-021-00628-0

**Published:** 2021-06-08

**Authors:** Yin Cao, Lingyun Liu, Jing Lin, Penghao Sun, Kaimin Guo, Shengqiang Li, Xian Li, Zi-jian Lan, Hongliang Wang, Zhenmin Lei

**Affiliations:** 1 Department of Andrology, the First Hospital of Jilin University, Changchun, Jilin 130021 People’s Republic of China; 2 Department of OB/GYN and Women’s Health, MDR Building, University of Louisville School of Medicine, 511 South Floyd Street, Louisville, KY 40292 USA; 3 Fujian Academy of Traditional Chinese Medicine, Fuzhou, 350003 China; 4 Present Address: Division of Life Sciences, Alltech, Nicholasville, KY 40356 USA

**Keywords:** Numb/Numb-like, Notch, FGF4, FGFR, Rete testis, Germ cells

## Abstract

Numb (Nb) and Numb-like (Nbl) are functionally redundant adaptor proteins that critically regulate cell fate and morphogenesis in a variety of organs. We selectively deleted *Nb* and *Nbl* in testicular germ cells by breeding *Nb/Nbl* floxed mice with a transgenic mouse line *Tex101-Cre*. The mutant mice developed unilateral or bilateral cystic dilation in the rete testis (RT). Dye trace indicated partial blockages in the testicular hilum. Morphological and immunohistochemical evaluations revealed that the lining epithelium of the cysts possessed similar characteristics of RT epithelium, suggesting that the cyst originated from dilation of the RT lumen. Spermatogenesis and the efferent ducts were unaffected. In comparisons of isolated germ cells from mutants to control mice, the Notch activity considerably increased and the expression of Notch target gene *Hey1* significantly elevated. Further studies identified that germ cell *Fgf4* expression negatively correlated the Notch activity and demonstrated that blockade of FGF receptors mediated FGF4 signaling induced enlargement of the RT lumen in vitro. The crucial role of the FGF4 signaling in modulation of RT development was verified by the selective germ cell *Fgf4* ablation, which displayed a phenotype similar to that of germ cell *Nb/Nbl* null mutant males. These findings indicate that aberrant over-activation of the Notch signaling in germ cells due to *Nb/Nbl* abrogation impairs the RT development, which is through the suppressing germ cell *Fgf4* expression. The present study uncovers the presence of a lumicrine signal pathway in which secreted/diffusible protein FGF4 produced by germ cells is essential for normal RT development.

**Supplementary Information:**

The online version contains supplementary material available at 10.1007/s12079-021-00628-0.

## Introduction

Numb (Nb) and its homolog Numb-like (Nbl) in mammals are functionally redundant intracellular adaptor proteins that critically regulate cell fate choice, cell–cell adhesion, and morphogenesis in a variety of tissues and organs. Antagonizing Notch signaling is one of well characterized Nb/Nbl mediated actions in regulation of these cellular and developmental processes (Gulino et al. 2010; Katoh and Katoh 2006; Shao et al. 2017; Verdi et al. 1996; Wahi et al. 2016; Zhang et al. 2016). In mice, there are four Notch receptor proteins (Notch1—4) and two families of ligands, Delta-like (Dll1—4), and Jagged (Jag1 and 2) (Artavanis-Tsakonas and Muskavitch 2010; Lai 2004). Notch receptors are activated upon the binding of the ligands from neighboring cells and triggers a γ-secretase-dependent proteolytic cleavage within the transmembrane domain to release the Notch intracellular domain (NICD). NICD is transported into the nucleus, where NICD acts as a coactivator with other transcription factors to regulate the expression of downstream target genes, including hairy and enhancer of split (*Hes*) and Hes related with YRPW motif (*Hey*), two families of transcription repressors that modulate a number of genes to influence multiple cellular processes in response to ligand-induced activation of all Notch proteins (Jarriault et al. 1995; Kopan and Ilagan 2009; Schroeter et al. 1998; Wahi et al. 2016). Nb/Nbl-dependent inhibition of Notch signaling is mediated by targeting activated Notch NICD to polyubiquitination and degradation as well as stimulating endocytosis and sequestration of pre-cleaved Notch or membrane tethered NICD (Berdnik et al. 2002; McGill and McGlade 2003; Shao et al. 2017).

Nb/Nbl and core components of the Notch signaling pathway express in all cell lineages starting from early gonadogenesis in mammals (Corallini et al. 2006; Hahn et al. 2009; Hayashi et al. 2004; Jameson et al. 2012; Lin et al. 2017; Murta et al. 2013; von Schonfeldt et al. 2004). Complete ablation of *Nb/Nbl* results in early embryonic lethality in mice (Petersen et al. 2002; Zhong et al. 2000; Zilian et al. 2001). Conditional deletion of *Nb* on an *Nbl* mutant background reveals that Nb/Nbl are important for somatic cell lineage commitment through the repression of Notch signaling activity in the early fetal development of mammalian testis (Lin et al. 2017). It is reported that the Notch signaling plays a pivotal role in germ cell development in flies, nematodes and Xenopus (Assa-Kunik et al. 2007; Kimble and Crittenden 2007; Morichika et al. 2010). Although Notch receptors and their ligands as well as Nb/Nbl express in somatic cells and germ cells at different steps of differentiation in adult murine testis, testicular Notch signaling in regulation of germ cell function and spermatogenesis in mammals are controversial. Some studies demonstrated that blockade of Notch receptors by antibodies or by a chemical inhibitor in vitro or in vivo impaired spermatogenesis, indicating its necessity for the maintenance of germ cell survival and differentiation (Murta et al. 2014). By contrast, mutant mice with constitutively active Notch NICD in Sertoli cells showed aberrant migration, premature differentiation of gonocytes and germ cell loss (Garcia et al. 2013). Mice with germ cell-specific overexpression of NICD exhibited an increase of germ cell apoptosis of aged animals, suggesting aberrant activation of the Notch signaling could lead to defective spermatogenesis (Huang et al. 2013). Loss-of-function studies, on the other hand, showed that Notch signaling was not essential for normal spermatogenesis. Meanwhile, histology of seminiferous tubules in mice with deletion of Notch1 and Pofut1, a fucosyltransferase that activates all Notch receptors by transferring fucose to the Notch extracellular domain, displayed no discernible spermatogenic deficiency (Batista et al. 2012; Hasegawa et al. 2012). Furthermore, phenotypic analyses of transgenic mice carrying an active Notch NICD showed no effect on spermatogenesis but exhibited cystic dilation of the rete testis (RT) (Lupien et al. 2006). Interestingly, the phenotype of cystic dilation of the RT with normal spermatogenesis closely resembles that of lunatic fringe (Lfng) inactivated male mice (Hahn et al. 2009). Lfng is a glycosyltransferase known to positively or negatively modify Notch activity via the glycosylation of Notch receptors in a ligand-dependent manner (Fleming et al. 1997; Kakuda and Haltiwanger 2017; Klein and Arias 1998; Okubo et al. 2012; Panin et al. 1997). These findings suggest that dysregulated Notch signaling activity in reproductive tissue might disturb the development of the RT.

Cross-talk between the Notch and fibroblast growth factor (FGF) signaling pathways plays important roles in modulating organogenesis, cell differentiation and migration (Bongarzone et al. 2000; Faux et al. 2001; Katoh and Katoh 2006; Miralles et al. 2006; Small et al. 2003; Wei et al. 2019). FGFs are a large family of structurally related, widely expressed, multifunctional signaling proteins. The signals aroused by FGFs are converted primarily by four FGF receptors (FGFRs), namely FGFR1 to FGFR4, to exert plethora biological effects on embryonic development and homeostasis in the adult for a broad range of tissues (Cotton et al. 2008; Eswarakumar et al. 2005; Katoh and Nakagama 2014). Numerous FGFs including FGF4 and all four FGFRs express in some kind of cells in the male reproductive tract (Cancilla and Risbridger 1998; Jiang et al. 2013; Kitadate et al. 2019; Li et al. 2014; Yamamoto et al. 2000). Yamamoto et al. reported that the expression of FGF4 primarily localized in Sertoli cells and lower levels in interstitial and germ cells in adult murine testes (Yamamoto et al. 2000). A recent study described that FGF4 mainly expressed, similar to FGF5, in lymphatic endothelial cells and some other interstitial cells in murine testicular interstitium (Kitadate et al. 2019). FGF4 binds and activates FGFR1, FGFR2 and FGFR3 to exert either cell autonomous or non-cell autonomous effects on various tissues (Cotton et al. 2008; Kosaka et al. 2009; Ornitz et al. 1996). It has been shown that over expression of *Fgf4* in the seminiferous tubules of murine testes enhances spermatogenesis and germ cell survival (Yamamoto et al. 2002). FGF4 is present in the RT fluid and is thought to be a lumicrine factor (Cotton et al. 2008; Hinton et al. 1998). However, its roles in regulation of the development and/or functions of the RT and downstream reproductive tract are not yet clear.

The RT is a structure of interconnected anastomotic cavities and delicate tubules located in an area close to the cranial pole of the testis. It connects seminiferous tubules on the upstream side via a short transition structure called tubuli recti and links to efferent ducts on the downstream side. The lumen of the RT lines with a simple cuboidal epithelium. Its functions are thought to transport spermatozoa from the seminiferous tubules to the efferent ducts and to provide a site for fluid reabsorption (Beu et al. 2003; Dym 1976). The embryonic development of the RT in mammals is not well understood. Combes et al. provided evidence that the RT initially forms as a flattened perforated interconnection between the mesonephric tubules and the testicular cords at approximately E15.5 and extensive remodeling occurs postnatally in mice (Combes et al. 2009). It is postulated that the epithelial cells of the RT are modified Sertoli cells (Wrobel 2000). A recent study showed that the RT expressed a marker for gonadal somatic cells steroidogenic factor 1 (SF1) (Omotehara et al. 2020). The authors suggested that these SF1-expressing gonadal cells, which were slightly different from Sertoli cells, contributed to the RT (Combes et al. 2009; de Mello Santos and Hinton 2019; Kulibin and Malolina 2020). These findings are consistent with previous results that the epithelia of the adult and immature RT possess Sertoli-like cells (Kulibin and Malolina 2020; Malolina and Kulibin 2019). These cells express not only Sertoli cell proteins, including SRY-box transcription factor 9 (SOX9), Wilms tumor protein (WT1), GATA binding protein 4 (GATA4), Doublesex and Mab-3 related transcription factor 1 (DMRT1), Androgen receptor (AR) and SF1 but also markers of the RT, such as Paired box gene-8 protein (PAX8) and E-Cadherin (CDH1) (Combes et al. 2009; Kulibin and Malolina 2020; Magers et al. 2016; Malolina and Kulibin 2019; Wang et al. 2013).

Based on the expression of Notch signaling pathway components and downstream targets in the testis, we investigated whether Notch signaling is involved in postnatal testis development and spermatogenesis by analyzing the testes in which Notch signaling antagonists *Nb/Nbl* were selectively abrogated in germ cells. Our findings indicate that postnatal germ cell-specific deletion of *Nb/Nbl* results in a cystic dilation of the RT but has no effect on spermatogenesis, while Notch signaling activity is aberrantly elevated. Further studies demonstrate that the dysregulated Notch signaling significantly suppresses the expression of FGF4 specifically in testicular germ cells. The privation of this secreted/diffusible growth factor from germ cells contributes to the cystic dilation of the RT phenotype.

## Material and methods

### Animals

*Tex101-iCre* (*Tex-Cre*) mice, a transgenic *Cre* mouse line expressing an improved *Cre* recombinase driven by the mouse *Tex101* promoter in germ cells within the seminiferous tubules of the testis, were established previously in our laboratory (Lei et al. 2010). The floxed *Numb/Numb-like* (*Nb*^*f/f*^*/Nbl*^*f/f*^) mice (Wilson et al. 2007) were purchased from Jackson Laboratories (Bar Harbor, ME, USA). The floxed *Fgf4 (Fgf4*^*f/f*^*)* mice (Sun et al. 2000) were generously provided by Dr. Gail Martin (University of California San Francisco, CA, USA). For germ cell selective deletion of *Nb/Nbl* and *Fgf4*, *Tex-Cre* female mice were first bred with *Nb*^*f/*+^*/Nbl*^*f/*+^ or *Fgf4*^*f/*+^ males to obtain bi-genic heterozygous females (*i.e., Tex-Cre:Nb*^*f/*+^*/Nbl*^*f/*+^ and *Tex-Cre:Fgf4*^*f/*+^). Then, these females bred with *Nb*^*f/f*^*/Nbl*^*f/f*^ or *Fgf4*^*f/f*^ males to generate male germ cell-specific *Nb/Nbl* and *Fgf4* mutant mouse lines (male *Tex-Cre*:*Nb*^*f/f*^*/Nbl*^*f/f*^ or *Tex-Cre*:*Fgf4*^*f/f*^).

Mouse tail genomic DNA was extracted using Extracta™ DNA Prep for PCR-Tissue kit (Quantabio, Beverly, MA, USA) as described previously (Li et al. 2014). *Tex-Cre:Nb*^*f/f*^*/Nbl*^*f/f*^ and *Tex-Cre:Fgf4*^*f/f*^ mice were genotyped by PCR analysis using primers listed in supplemental table 1. The primer sets *Cre*, *Nb*^*Δ*^, *Nbl*^*Δ*^, and *Fgf4*^*Δ*^ were used to determine the deletion of floxed *Nb, Nbl* and *Fgf4* alleles and the presence of *Cre*.

Timed pregnancy was performed by placing 8 to 12 week-old females with males overnight, and vaginal plugs were checked the next morning. Fertilization was assumed to occur at midnight, and the time of plug identification was considered as embryonic day 0.5 (E0.5). The day of birth was defined as postnatal day 1 (D1).

All animals were kept on a 12-h light–dark cycle, with free access to food and water, in the vivarium of the University of Louisville. Other animal cares were maintained as required under the National Institutes of Health guide for the Care and Use of Laboratory Animals. All animal studies have been approved by the Animal Care and Use Committee at the University of Louisville. All mice were sacrificed under ketamine anesthesia and all efforts were made to minimize their suffering.

### Tracer dye studies

The area of the RT located at the vascular pole was exposed in 3 month-old *Tex-Cre* and *Tex-Cre:Nb*^f/f^/*Nbl*^f/f^ mice. A 0.25% solution of Evans blue (Sigma, St. Louis, MO, USA) was slowly injected into the rete testis using pulled glass needles as described by Dym (Dym 1976).The blue dye flowed into the lumen of seminiferous tubules, the efferent ducts and the epididymis of mice in *Tex-Cre* mice, indicating a successful injection in these non-cystic testes.

### Preparation of testicular cells

Testicular cells were isolated from prepubertal and adult mice using the procedure as reported previously (Li et al. 2014; Lin et al. 2014). Briefly, harvested testes were decapsulated. Dispersed seminiferous tubules were minced into small fragments and incubated in Hank's balanced salt solution (HBSS, Sigma) containing collagenase type I (0.5 mg/mL) in a water bath at 33^o^ C with constant shaking to separate interstitial cells from the seminiferous tubules. The seminiferous tubular fragments were settled down by unit gravity. The supernatant that contained interstitial cells was pelleted by centrifugation. The sedimentary seminiferous cord fragments were digested in HBSS containing trypsin (0.5 mg/mL) and DNase (1 μg/mL, Sigma) for 15 min in a water bath at 33^o^ C with constant shaking. The supernatant that contained germ cells were filtered through 40-µm nylon mesh (Santa Cruz Biotech, Dallas, TX, USA) and collected by centrifugation. The germ cells were then washed with Dulbecco’s modified eagle medium (DMEM, Sigma) twice and used for subsequent experiments. The precipitated cell pellet by unit gravity that contained Sertoli cells was rinsed with HBSS twice and cultured with DMEM supplemented with 10% fetal bovine serum (FBS, Sigma) overnight. Sertoli cells were harvested the next day after residual germ cells were hypotonically removed. The purity of isolated interstitial, Sertoli and germ cells was verified by performing reverse transcription-polymerase chain reaction (RT-PCR) using several marker genes for each cell type as described in our previous report (Lin et al. 2014). The results showed that the contamination of each cell type by the others was neglectable (data not shown).

### Cell culture

Murine spermatogonia cell line GC1-spg (GC1) was purchased from the American Type Culture Collection (Manassas, VA, USA). GC1 and germ cells purified from prepubertal testes were maintained in DMEM containing 10% FBS and 1% penicillin–streptomycin (Sigma) in a humidified atmosphere of 5% CO_2_ at 37^o^ C.

On the day of treatments of N-[N-(3,5-difluorophenacetyl)-l-alanyl]-S-phenyl glycine t-butyl ester (DAPT, Selleckchem, Houston, TX, USA), a γ-secretase inhibitor that suppressed all pathways of the Notch signaling, the medium was changed to phenol red- and serum-free DMEM. DAPT was dissolved in Dimethyl sulfoxide (DMSO, Sigma) and added to GC1 and isolated germ cells at the final concentrations of 5, 10, 20 and 30 µM, respectively. DMSO alone was used as the control.

To overexpress NICD in GC1 cells, the cells were cultured to approximately 80% confluence in 12-well plates and were transiently transfected with 1 µg of *p3XFlag-NICD* plasmid DNA (Ong et al. 2006) (Addgene, Watertown, MA, USA) with 2 µL of X-TremeGene 360 reagent (Roche, Mannheim, Germany) per well for 48 to 72 h. The cells were then cultured in DMEM containing 10% FBS. The cells transfected with vector *p3XFLAG-VMC* or treated with X-TremeGene 360 reagent alone served as controls. The overexpression of NICD protein levels in these cells was determined by Western blot.

### RNA extraction and RT-PCR analysis

Total RNA was isolated from the cells and testis tissues using Trizol reagent (Invitrogen, Carisbad, CA, USA). The integrity of the extracted total RNA was verified by agarose gel (0.8%) electrophoresis. The purity and concentration of extracted RNA were determined by a SpectroStar nano absorbance microplate reader (BMG Labtech, Cary, NC, USA). Subsequently, 2 µg of total RNA was reverse transcribed into cDNA using a high-capacity cDNA reverse transcription kit (Applied Biosystems, Foster, CA, USA) according to the protocol recommended by the manufacturer.

For semiquantitative PCR, the cDNA was amplified by PCR with the primer sets of the target genes and a housekeeping gene ribosomal protein large subunit 19 (*Rpl19*) as listed in supplemental table 1. The Amplification was performed on a BioRad iCycler with the following reaction conditions: initial denaturation at 98 °C for 3 min followed by 34 cycles of 30 s at 94 °C, 30 s at 58 °C and 1 min at 72 °C, and a last extension step of 7 min at 72 °C. The amplified products were separated by agarose gel electrophoresis and stained with ethidium bromide (Sigma). The intensity of specific bands was scanned and semi-quantified using the image analysis software, TotalLab V (Nonlinear USA Inc, Durham, NC). The results were presented as the ratio of target gene over *Rpl19*.

For real-time PCR, cDNA was amplified with primer sets of target genes as listed in supplemental table 1 and Power Track SYBR Green master mix (Thermo Fisher Scientific, Waltham, MA, USA) on a StepOne plus thermal cycler (Applied Biosystems). The PCR was carried out with the following cycling parameters: 2 min at 95 °C followed by 40 cycles of 15 s at 95 °C and 1 min at 60 °C. Data were normalized to a housekeeping gene *Rpl13* and the fold change of the target gene expression levels were calculated using the ^ΔΔ^Ct method.

All the oligonucleotide primers listed in supplemental table 1 were designed according to the sequences obtained from GenBank using the Vector NTI 12.0 program (Invitrogen) and synthesized by Operon Technologies (Alameda, CA, USA).

### Protein isolation and Western blot analysis

GC1 cells and the purified testicular germ cells were washed twice with ice-cold phosphate buffered saline (PBS) and lysed with radioimmunoprecipitation (RIPA) lysis buffer (Sigma) on ice. For mouse testis tissue, after being decapsulated and minced into small pieces, the seminiferous tubules were homogenized in RIPA lysis buffer using sonic dismembrator (Fisher Scientific) at 4 °C. The protein concentrations in supernatants were determined by a bicinchoninic acid protein assay kit (Thermo Fisher Scientific). Subsequently, 14 µg of protein per sample were boiled at 95 °C for 5 min, separated on sodium dodecyl sulphate–polyacrylamide gel electrophoresis and transferred to poly-vinylidene fluoride membranes (Immobilon, St. Louis, MO, USA). After being blocked with 3% non-fat milk (Bio-Rad Laboratories) in PBS for 1 h at room temperature, the membranes were incubated overnight at 4 °C with the primary antibodies as listed in supplemental table 2. The membranes were washed in Tris-buffered saline containing 0.1% tween-20, incubated with horseradish peroxidase-linked secondary antibody and detected by Clarity™ Western enhanced chemiluminescence substrate (Bio-Rad Laboratories). The membranes were re-blotted with β-actin or β-tubulin which served as a loading control. The molecular size of proteins was determined by running marker protein standards in an adjacent lane. Blots were quantified by measuring the intensity from correctly sized bands under ToalLab V (Nonlinear USA Inc). The optical density of protein bands was normalized by β-tubulin or β-actin.

### Testicular explant culture

The procedure of testicular explants was performed as Kojima et al. described previously (Kojima et al. 2016; Sato et al. 2013). Briefly, 1.5% agarose gel was prepared with low melting agarose (FMC Bio Products, Rockland, ME, USA). One day before testis culture, the agarose gel was cut into small pieces (approximately 1 cm in diameter and 5 mm height) and submerged in the culture medium. The culture medium was made with 100 mL of DMEM including 10 mL of knockout serum and 1 mL of penicillin–streptomycin (Invitrogen). The testes were dissected from P1 male pups. Each testis was placed on the top of a medium presoaked agarose stand in a 35-mm dish. The medium was added to the dish to reach about four-fifths height of agarose stand without covering the testis and cultured in a humidified atmosphere of 5% CO_2_ at 34 °C overnight. The testes were then treated with the following reagents for 72 h. (1). Recombinant FGF4 (Proteintech, Rosemont, IL, USA) at the final concentrations of 10, 25 and 50 ng/mL, respectively. FGF4 was reconstituted in PBS containing 0.1% endotoxin-free recombinant human serum albumin (HumanZyme, Hamburg, Germany). (2). LY2874455 (LY, Selleckchem), a pan-FGF receptor (FGFR) inhibitor, was prepared in DMSO and added to the culture medium at final concentrations of 0.1, 0.5 and 1 µM, respectively. (3). Co-treatments of 10, 25 and 50 ng/mL recombinant FGF4 and 0.5 µM LY. (4). BLU9931 (BLU; MedKoo Biosciences, Morrisville, NC, USA), a selective FGFR4 inhibitor, was dissolved in DMSO and added to the culture medium at final concentrations of 0.25 and 0.5 µM, respectively. (5). The vehicles PBS and DMSO were used as controls.

### Histological and immunohistochemical analyses

Testes were fixed in 10% formalin overnight. The orientation of the testes was kept the same for all the testes in HistoGel (American MasterTech, Lodi, CA, USA) before embedded in paraffin. Longitudinal serial sections (5 µm) for each sample were cut. Every five sections were deparaffinized, rehydrated and stained by hematoxylin and eosin (H&E) for general morphological observation. The testicular section with the greatest long-axis and largest area for each sample was used for morphometric analyses.

For immunohistochemical staining, all sections underwent antigen retrieval by microwave heating in 0.01 M citrate buffer (pH 6.0) for 13 min. Slides were washed in PBS followed by incubation in 0.3% H_2_O_2_ to deactivate endogenous peroxidase activity and then nonspecific binding was blocked with 5% normal goat serum for 1 h at room temperature. Sections were then incubated with the primary antibodies as listed in supplemental table 2 at 4 °C in a humidified chamber overnight. After being washed three times with PBS, the slides were incubated with 1:100 diluted goat anti-rabbit or goat anti-mouse biotinylated secondary antibody for 1 h followed by incubation in a solution of avidin–biotin peroxidase complex for 1 h at room temperature. Immunoreactivity was detected by incubation of the sections with the substrate 3′3-diaminobenzidine (Vector Lab, Burlingame, CA, USA). The slides were counterstained with hematoxylin and visualized by an Olympus IX71 light microscope (Olympus Corp, Center Valley, PA). Replacement of the primary antibody with PBS was performed at the same time as a procedure control. We also used irrelevant rabbit IgG instead of the primary antibody to check the specificity of the immunostaining.

The digital images were captured and quantitatively analyzed using Stream Image analysis software (Olympus Corp). The RT area was defined by the morphology of H&E staining and confirmed by PAX8 immunostaining. The percentage of DMRT1 immunostained positive cells was determined by counting all the epithelial cells of the RT from one representative section. The luminal area of the RT was measured from three longitudinal consecutive H&E stained sections through midline per sample. All samples were independently examined by two investigators who were blind to the experimental information.

### Statistical analysis

Statistical analysis was performed with Graph Pad Prism 8.0 (Graph Pad Software, San Diego, CA, USA). All assays were performed at least three independent replicates. The data presented are the means ± SEM. All results were analyzed by independent *t *test for the comparison between two groups. Means of more than two groups were compared using one-way ANOVA. A *p *value < 0.05 was considered statistically significant.

## Results

### Selective inactivation of *Nb/Nbl* in testicular germ cells leads to cystic dilation of the RT

**Fig. 1:**
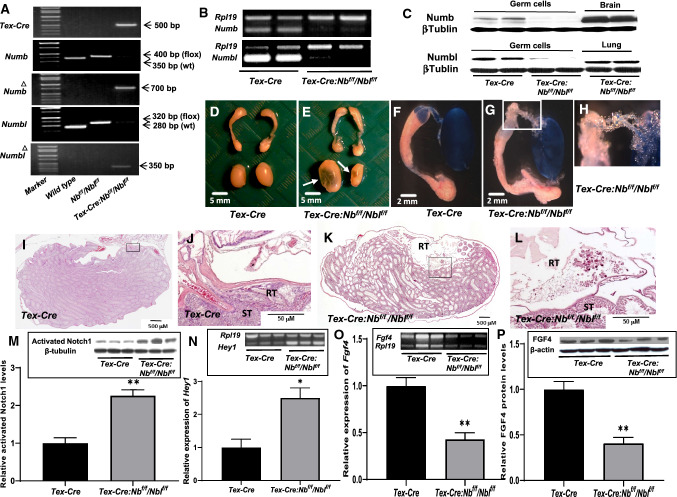
Testicular germ cell-specific deletion of *Numb/Numbl* (*Nb/Nbl*) results in cystic dilation of the RT, aberrant activation of Notch signaling and decrease in *Fgf4* expression. Excision of the floxed *Nb/Nbl* alleles by*Tex-Cre* in testicular germ cells. Representative PCR genotyping results (**a**) show the lack of the *Nb/Nbl* alleles and the presence of the *Nb*^*∆*^*/Nbl*^*∆*^ alleles indicating the deletion of the floxed *Nb/Nbl* alleles in the presence of *Cre* transgene in *Tex-Cre:Nb*^*f/f*^*/Nbl*^*f/f*^ mice. RT-PCR and Western blot demonstrate the absence of *Nb/Nbl* mRNA (**b**) and proteins (**c**) in isolated testicular germ cells. The brain and lung (**c**) serve as positive control tissues for Nb and Nbl, respectively. Gross appearance of testes and epididymis of 3 month-old *Tex-Cre* (**d**) and *Tex-Cre:Nb*^*f/f*^*/Nbl*^*f/f*^ (**e**) mice. Bilateral cysts are indicated with arrows. Injection of Even blue into the lumen of the RT of *Tex-Cre* (**f**) and *Tex-Cre:Nb*^*f/f*^*/Nbl*^*f/f*^ (**g**) mice. H is a magnified image of boxed area in G showing that tracer dye is hardly seen in the efferent ducts. Longitudinal section through midline of a 3-month-old *Tex-Cre* testis (**i**, **j**). H&E staining shows that the RT is located at the cranial pole of the testis and the union of the RT and the efferent ducts. Longitudinal section through midline of a 3-month-old*Tex-Cre:Nb*^*f/f*^*/Nbl*^*f/f*^ testis (**k**, **l**). A dilated lumen at the testicular hilum containing debris of dead spermatocytes is shown. J and L are magnified images of boxed areas in I and K, respectively. Deletion of *Nb/Nbl* significantly increases activated Notch1 NICD protein levels (**m**) and induces high expression of a Notch target gene *Hey1* (**n**) in testicular germ cells. RT-PCR and Western blot results show that *Fgf4* mRNA (**o**) and FGF4 protein (**p**) levels in testicular germ cells are significantly reduced in *Tex-Cre:Nb*^*f/f*^*/Nbl*^*f/f*^ (n = 3) compared to *Tex-Cre* siblings (n = 3). Statistical analyses are performed by *t *test. *p < 0.05, **p < 0.01 and ***p < 0.001

For germ cell-selective deletion of *Nb/Nbl*, *Tex-Cre* mice were bred with *Nb*^*f/f*^*/Nbl*^*f/f*^ mice to generate *Tex-Cre:Nb*^*f/f*^*/Nbl*^*f/f*^ male offspring. Genotyping analysis of the progenies was performed. PCR results showed the lack of the *Nb/Nbl* alleles and the presence of the *Nb*^*Δ*^*/Nbl*^*Δ*^ alleles indicating the deletion of the floxed *Nb/Nbl* alleles in the presence of *Cre* transgene in these mice, indicating complete deletion of the floxed *Nb/Nbl* alleles in the male germline (Fig. 1a). Deletion of *Nb/Nbl* in the germ cells of *Tex-Cre:Nb*^*f/f*^*/Nb*^*f/f*^ males was further confirmed by performing RT-PCR and Western blot. The results showed that the *Nb/Nbl* transcripts (Fig. 1b) and Nb/Nbl proteins (Fig. 1c) in testicular germ cells of *Tex-Cre:Nb*^*f/f*^*/Nbl*^*f/f*^ animals were not detectable. In congruence with the results reported by Corallini et al. (Corallini et al. 2006), immunohistochemical staining of testicular sections showed that Nb was found in somatic (Sertoli and interstitial cells) and all groups of germ cells in the adult *Tex-Cre* testis (Suppl. Figure 1A). Nbl exhibited the same immunostaining pattern as Nb (data not shown). In three month-old *Tex-Cre:Nb*^*f/f*^*/Nbl*^*f/f*^ testis, immunostaining of Nb (Suppl. Figure 1C & D) and Nbl (Suppl. Figure 1E & F) was detected in testicular somatic cells the same as in the adult *Tex-Cre* testis. However, they were absent in the germ cells.

The testes of *Tex-Cre:Nb*^*f/f*^*/Nbl*^*f/f*^ three month-old male mice developed unilateral or bilateral cysts at the testicular hilum. There were no gross abnormalities and size difference in the epididymis (Fig. 1d–h) and other accessary sex organs (data not shown) in *Nb/Nbl* mutant male mice. Injection of Even blue into the RT of mutant mice demonstrated partial or complete blockages in the RT as evidenced by lack of, or very faint, blue dye in the efferent ducts and caput epididymis (Fig. 1d–h). Histological and immunohistochemical examination (Fig. 1i–l, Suppl. Figure 1A – D & Fig. 5i–k) revealed that the lining epithelium of the cysts possessed similar characteristics of RT epithelium. Unlike normal RT in *Tex-Cre* mice, which forms anastomotic channels limited to an area close to the cranial pole of the testis and connected to the seminiferous tubules via a short transition region called tubuli recti. The RT was irregularly distributed in mutant mice. The cystic dilated RT contained degenerative spermatocytes and spermatozoa (Fig. 1i–l). Degeneration of spermatocytes and spermatozoa may be caused by chronically trapped in dilated cysts.

As the cysts, which were clearly observed around postnatal 20 to 25 days, grew in size with age, they compressed the surrounding and nearby seminiferous tubules. Under the conditions of routine tissue processing (*i.e.* fixation with 10% formalin, dehydration with ethanol and embedding with paraffin), the appearance of loosening interstitial space was more commonly observed in the cyst-containing testes than the testes without cysts. The epithelium of these squeezed seminiferous tubules appears to be thinner than the ones that were not close to the dilatated cyst. Otherwise, the histological structures of efferent ducts and the size of the seminiferous tubules of *Tex-Cre* and *Tex-Cre:Nb*^*f/f*^*/Nbl*^*f/f*^ (Suppl. Figure 2 K & L) were essentially indistinguishable. Light microscopy revealed that in *Tex-Cre* and mutant mice, all stages of spermatogenesis were present (Suppl. Figure 2A–D). We detected no significant changes in GCNF-positive spermatogenic cells, GATA4-positive Sertoli cells and Cyp17A1-positive interstitial cells in these mutant testes (Suppl. Figure 2E–J). Although thorough studies were not performed on female mice, no abnormalities were noted in the ovaries, fallopian tubes and uterus for any of the investigated genotypes (data not shown).

### Activation of the Notch signaling and reduction of *Fgf4* expression in absence of *Nb/Nbl* in testicular germ cells

It is well established that Nb and Nbl are negative regulators of Notch signaling (Shao et al. 2017; Zhang et al. 2016). To examine the activity of Notch signaling in testicular germ cells of *Tex-Cre:Nb*^*f/f*^*/Nbl*^*f/f*^ mice, we used a specific antibody for the cleaved Notch intracellular domain NICD1. Western blot analysis showed that cleaved NICD1 protein levels were significantly elevated in the germ cells of *Tex-Cre:Nb*^*f/f*^*/Nbl*^*f/f*^ testes (Fig. 1m). Moreover, *Hey1*, a well-known target gene of the Notch signaling, was markedly upregulated in the germ cells lacking *Nb/Nbl* (Fig. 1n). In contrast, the expression of *Fgf4* and FGF4 protein in these germ cells were significantly reduced in *Tex-Cre:Nb*^*f/f*^*/Nbl*^*f/f*^ mice compared to *Tex-Cre* siblings (Fig. 1o, p).

### The activity of Notch inversely correlates with *Fgf4* expression in testicular germ cells

**Fig. 2:**
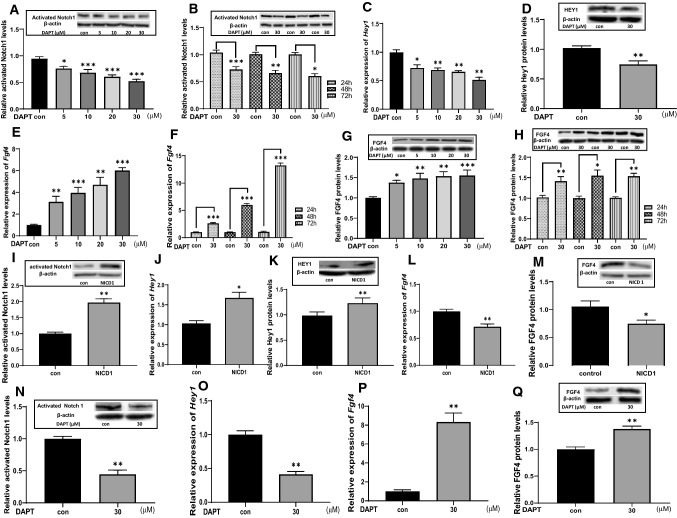
Effects of Notch activity on *Fgf4* expression in GC1 and purified testicular germ cells. Dose (**a**) and time (**b**) dependent inhibition of Notch activity by DAPT in GC1 cells, which also results in a significant decrease of Notch target gene *Hey1* mRNA (**c**) and HEY1 protein (**d**) levels. Dose (**e**, **g**) and time (**f**, **h**) dependent elevation of *Fgf4* mRNA (**e**, **f**) and FGF4 protein levels (**g**, **h**) by DAPT in GC1 cells. Over-expression of NICD in GC1 cells (**i**) induces *Hey1* mRNA (**j**) and HEY1 protein (**k**) levels, while *Fgf4* mRNA (**l**) and FGF4 protein (**m**) levels are significantly reduced. Suppression of Notch activity by DAPT in primarily cultured testicular germ cells isolated from pre-pubertal mice (**n**) results a reduction of *Hey1* mRNA levels (**o**), but induces higher *Fgf4* mRNA (**p**) and FGF4 protein (**q**) levels. All the mRNA levels are determined by real-time PCR and all the protein levels are detected by Western blot (n = 5). One-way ANOVA is performed for A, C, E and G and the others are analyzed by *t *test. *p < 0.05, **p < 0.01 and ***p < 0.001 compared to controls

We first used murine germ cell line GC1 to examine whether *Fgf4* expression is modulated in response to the activity of the Notch signaling. Western blot results showed a time- and dose-dependent suppression of activated Notch by DAPT in GC1 cells with a maximum inhibition of 30 µM for 48 h of incubation (Fig. 2a, b). Meanwhile, the mRNA and protein levels of the Notch target gene *Hey1* were significantly reduced in parallel with the activity of the Notch signaling (Fig. 2c, d). In contrast, when GC1 cells were treated with DAPT at the same conditions, *Fgf4* expression was increased in a dose- and time-dependent manner (Fig. 2e, h). To further characterize Notch-dependent suppression of *Fgf4* expression, we overexpressed NICD in GC1 cells which also trans-activated the *Hey1* expression (Fig. 2i–k), while the *Fgf4* mRNA and FGF4 protein levels strikingly declined (Fig. 2l, m). Furthermore, Notch-dependent modulation of *Fgf4* expression was further examined in primary cultured murine germ cells. RT-PCR results showed that *Fgf4* mRNAs were readily detectable in the whole testis as well as in purified interstitial, germ and Sertoli cells in adult mice (Suppl. Figure 3A). Analyses of *Fgf4* expression by RT-PCR during postnatal testicular development revealed that *Fgf4* mRNA levels in the testis rapidly increased during neonatal period and then gradually decreased from pubertal period to adulthood (Suppl. Figure 3B). Western blot results showed that FGF4 protein levels in the testis dramatically elevated during neonatal period and remained constant from neonatal period to adulthood. Immunohistochemical staining of testicular sections revealed that FGF4 (Suppl. Figure 3D) were detected in both the interstitial and seminiferous tubular compartments from neonatal to adulthood. The most prominent immunostaining of FGF4 was observed in interstitial cells. In the seminiferous tubular compartment, the immunostaining for FGF4 was evident in spermatocytes, elongated spermatid, spermatozoa and weak immunostaining was present in spermatogonia and Sertoli cells throughout all phases of postnatal development. Therefore, we isolated the germ cells from prepubertal testes and treated them with 30 µM DAPT for 48 h. The real-time PCR and Western blot results demonstrated that the mRNA and protein levels of FGF4 were markedly increased (Fig. 2n–q). Together, these genetic and pharmacological experiments suggest that constitutive activation of the Notch signaling represses *Fgf4* expression in testicular germ cells.

### Inhibition of FGFR induces RT dilation in explant-cultured testes

Preliminary experiments showed that there was no tissue degeneration observed in all testes from E13.5 to D1 for up to 4 days in culture. As defined by immunolabeling with GATA4 (both RT epithelium and Sertoli cells), DMRT1 (Sertoli cells and few cells scattered in the tubuli recti) and PAX8 (RT epithelium only), the RT with open cavities were distinctly identified in D1 testes. The size of RT cavities increased gradually with time indicating that the RT is continuing to grow in explant culture (Suppl. Figure 4). These results demonstrate the technical feasibility of using explant-cultured D1 testes to test whether FGF4/FGFR signaling influenced the RT development at the time that corresponds to the beginning of *Fgf4* deletion in *Tex-Cre:Fgf4*^*f/f*^ mice.

**Fig. 3:**
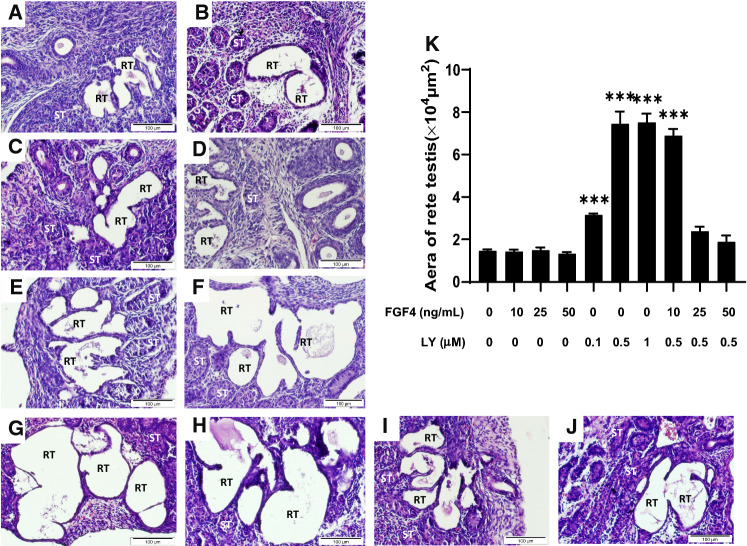
Inhibition of FGF signaling results in dilation of the rete testis (RT) in neonatal testicular explants. Representative images of H&E stained explant cultured P1 testicular sections treated with vehicle (**a**), various amounts of exogenous FGF4 [10 (**b**), 25 (**c**) and 50 (**d**) ng/mL], FGF receptor inhibitor LY 2,874,455 (LY) [0.1 (**e**), 0.5 (**f**) and 1 (**g**) µM] and co-treatments of various amounts of FGF4 [10 (**h**), 25 (**i**) and 50 (**j**) ng/mL] and 0.5 µM LY for 72 h. Histogram (K) show data of the areas of RT lumen from D1 testicular explants for all treatments (n = 3). Statistical analysis is performed by One-way ANOVA. ***p < 0.001 compared to controls. ST (Seminiferous tubule), RT (Rete testis)

H&E staining showed that the addition of various amounts of recombinant FGF4 had no visible effect on the morphology of the RT compared to the control group (Fig. 3a–d). The areas of the RT in both groups had no significant difference (Fig. 3k). This outcome may be reasonable to assume that due to sufficient endogenous FGF4, exogenous FGF4 would have little or no additive effect. On the other hand, treatment with a pan-FGFR inhibitor LY2874455 caused a remarkable enlargement of RT lumens (Fig. 3a, e–g). Morphometric analysis showed that the area of the RT in LY2874455 treated testes was significantly increased compared to the control group with the maximum induction at a dose of 0.5 µM (Fig. 3k). By contrast, treatment with a FGFR4 selective inhibitor BLU9931 exhibited little or no effect on the induction of the enlargement of RT lumen (data not shown). Interestingly, coincubation of FGF4 and LY2874455, the enlargement of RT lumens was markedly diminished. Supplement of 25 ng/mL of FGF4 completely antagonized the effect of LY2874455 on induction of RT luminal dilation (Fig. 3a, h–j, k), indicating that inhibition of FGF4/FGFR signaling activity impaired normal development of the RT.

### FGFR blockade increases DMRT1 positive epithelial cells in the RT

**Fig. 4:**
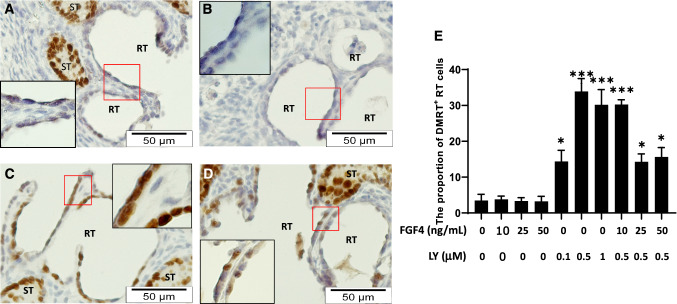
Effects of FGF4 and FGF receptor inhibitor on DMRT1 expression in RT cells. Representative DMRT1 immunostaining images of neonatal testicular explants treated with vehicle (**a**), 25 ng/mL FGF4 (**b**), 0.5 µM LY2874455 (LY, **c**) and co-treatment of 25 ng/mL FGF4 and 0.5 µM LY (**d**) for 72 h. The insets are magnified images of boxed areas in corresponding pictures. Histogram (**e**) Represent the percentage of DMRT1 positive nuclei in lining epithelium of rete testis (RT) from multiple neonatal testicular explants for all treatments (n = 5). Statistical analysis is performed by One-way ANOVA. *p < 0.05, ***p < 0.001 compared to controls. ST (seminiferous tubule), RT (rete testis)

To evaluate cellular mechanism by which FGF4/FGFR mediated effects on the development of the RT, we assessed the expression of several proteins in RT epithelial cells by immunohistochemical staining. DMRT1 was normally expressed in Sertoli cells and few cells in the tubuli recti, a short transition region between the seminiferous tubules and the RT, in neonatal testes. DMRT1 expression was not detected in RT epithelium (Suppl. Figure 4I–L). The distribution pattern and expression levels of DMRT1 did not change by the addition of exogenous FGF4 to the testis explants, while a significant increase of DMRT1 positive RT epithelial cells was observed by the addition of FGFR inhibitor LY2874455 in a dose dependent fashion (Fig. 4c, e). Treatments of testis explants with both exogenous FGF4 and LY2874455 together markedly reduced the number of DMRT1 positive RT epithelial cells compared to LY2874455 treatment alone (Fig. 4c–e). Immunohistochemical staining also demonstrated that GATA4, WT1, SF1, and SOX9 (Suppl. Figure 5) were readily detectable in Sertoli as well as RT epithelial cells, and PAX8, ESR1, DAX1 and CDH1 were localized in RT epithelial cells only (Suppl. Figure 5). However, there were no significant changes observed in these proteins with respect to their distribution pattern and immunostaining intensity in the testis explants treated with FGF4 or LY2874455 alone or in a combination of both (Suppl. Figure 5).

### Selective deletion of *Fgf4* in testicular germ cells results in cystic dilation of the RT

**Fig. 5:**
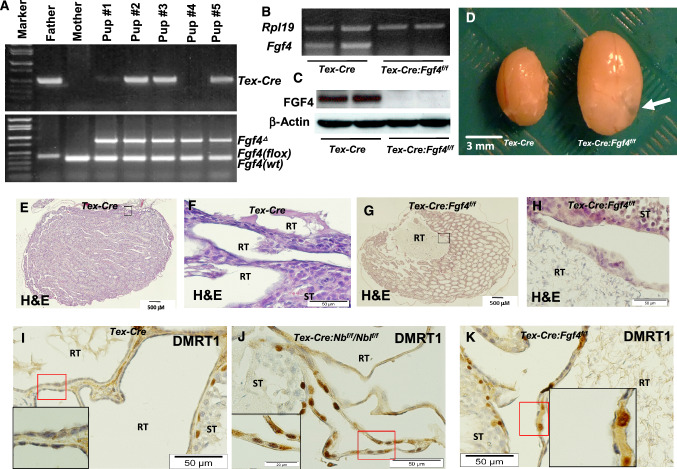
Testicular germ cell-specific deletion of *Fgf4* results in cystic dilation of the RT. Representative PCR genotyping results of a litter of pups from breeding of a *Tex-Cre:Fgf4*^*f/f*^ male with a wild type (WT) female (**a**). Note the lack of the *Fgf4* alleles and the presence of the *Fgf4*^*∆*^ alleles in all pups, indicating complete deletion of the floxed *Fgf4* alleles in the male germline, regardless of presence of *Cre* transgene in the progeny. RT-PCR and Western blot demonstrate the absence of *Fgf4* mRNA (**b**) and FGF4 protein (**c**) in purified testicular germ cells. Gross morphology of testes of 3 month-old *Tex-Cre* and *Tex-Cre:Fgf4*^*f/f*^ mice. A cystic RT of *Tex-Cre:Fgf4*^*f/f*^ testis is indicated by an arrow. H&E staining of the testes of 3-month-old *Tex-Cre* (**e**, **f**) and *Tex-Cre:Fgf4*^*f/f*^ (**g**, **h**) mice. **f**, **h** are magnified images of boxed areas in **e** and **g**, respectively. Numerous nuclei of RT epithelial cells of *Tex-Cre:Nb*^*f/f/*^*Nbl*^*f/f*^ (**j**) and *Tex-Cre:Fgf4*^*f/f*^ (**k**) testes are immunostained for DMRT1, while hardly any DMRT1 immunostained epithelial cells of the RT in *Tex-Cre* testis are observed (**i**). ST (seminiferous tubule), RT (rete testis)

To determine whether FGF4 produced by germ cells affect the development of the RT in vivo, we selectively deleted germ cell *Fgf4* in mouse by crossing *Tex-Cre* transgenic mice with floxed *Fgf4* mice. As shown in Fig. 5, genotyping PCR confirmed the floxed *Fgf4* alleles were excised (Fig. 5a). In the purified germ cells of *Tex-Cre:Fgf4*^*f/f*^ testes, RT-PCR analysis demonstrated that *Fgf4* mRNA was absent (Fig. 5b), and the FGF4 protein wasn’t detectable by Western blot (Fig. 5c), indicating that the *Fgf4* is efficiently deleted in germ cells by *Cre* recombinase.

It was noted that three month-old *Tex-Cre:Fgf4*^*f/f*^ males had unilateral or bilateral testicular cysts. The gross appearance of the testes of *Tex-Cre:Fgf4*^*f/f*^ mice was bigger than *Tex-Cre* mice due to the dilatation of the RT, as evidenced by the morphological study (Fig. 5d). Histological analysis showed that these testicular cysts were dilated RT and contained degenerated spermatocytes and spermatozoa (Fig. 5g, h). However, seminiferous tubules with a normal epithelium and no significant change in germ cell group observed in each seminiferous stage in the testes with cysts, suggesting spermatogenesis is not defective (Fig. 5g, h). Interestingly, immunohistochemical staining revealed that the same as in the testes of *Tex-Cre:Nb*^*f/f*^*/Nbl*^*f/f*^ mice (Fig. 5j), there were DMRT1 positive epithelial cells of cystic RT in T*ex-Cre:Fgf4*^*f/f*^ testes (Fig. 5k), while the epithelial cells of the RT in adult *Tex-Cre* testes were devoid of DMRT1 immunostaining (Fig. 5i). We detected no significant differences in immunostaining of aquaporin 3 (AQP3), AQP9 and cystic fibrosis transmembrane conductance regulator (CFTR) in the efferent ductule epithelium among *Tex-Cre*, *Tex-Cre:Nb*^*f/f*^*/Nbl*^*f/f*^ and *Tex-Cre:Fgf4*^*f/f*^ mice (Suppl. Figure 6). In the neonatal mouse efferent ductules, immunostaining for AQP3, AQP9 and CFTR were not observed in any treatment groups (data not shown). This is consistent with previous report that no AQP1 and AQP9 detectable by immunohistochemistry in rat efferent ductules before postnatal days 7 to 21 (Badran and Hermo 2002). It is possible that no expression of these proteins or too low to be detected by immunohistochemistry in neonatal murine efferent ductules.

## Discussion

To circumvent the early embryonic lethal phenotype of *Nb/Nbl* null mutation, we explored the role of Nb/Nbl specifically in testicular germ cells by deleting the exon 1 of *Nb* and the first 3 exons of *Nbl* within the seminiferous tubules, and subsequently breeding *Tex-Cre* transgenic mice and *Nb/Nbl–floxed* mice (*Nb*^*f/f*^*/Nbl*^*f/f*^). Surprisingly, the testes of *Tex-Cre:Nb*^*f/f*^*/Nbl*^*f/f*^ adult mice developed unilateral or bilateral cysts at the testicular hilum while the seminiferous tubules, efferent ductules and epididymis appear to be normal. Injection of trace dye into the RT of mutant mice demonstrated partial blockages in the RT as evidenced by very faint, blue dye in the efferent ducts and caput epididymis. Histological and immunohistochemical examination revealed that the lining epithelium of the cysts possessed similar characteristics of RT epithelium, suggesting that the cyst originated from dilation of the RT lumen.

Transgenic mice that constitutively expressed active Notch NICD driven by a mouse mammary tumor virus (MMTV) promoter displayed dilated lumen of the RT, while the spermatogenesis appeared to be normal in young adults (Lupien et al. 2006). The studies showed MMTV induced NICD overexpression in almost all of the male reproductive systems including germ cells (Huang et al. 2013; Lupien et al. 2006). Hahn et al. reported that global *Lfng* knockout in mice resulted in cystic dilation of the RT and again, spermatogenesis was unaffected in young adults (Hahn et al. 2009). Lfng is one of the three fringe proteins in mammals that acts cell-autonomously to inhibit Jag-dependent Notch activation or to potentiate Delta-dependent Notch activation (Fleming et al. 1997; Kakuda and Haltiwanger 2017; Klein and Arias 1998; Okubo et al. 2012; Panin et al. 1997). Since *Lfng* and *Jag1* predominately express in testicular germ cells (Hahn et al. 2005), it is not unreasonable to assume that the Notch signaling was likely to be enhanced in *Lfng* null germ cells. Together, these findings advocate the idea that dysregulation of Notch activity interferes with RT development. However, whether over activation of the Notch signaling in testicular germ cells leads to defective RT remains uncertain, although there were no reports that selective overexpression of NICD in Sertoli and/or Leydig cells induced cystic dilation of the RT (Ferguson et al. 2016; Garcia et al. 2013). The present study demonstrated that Notch activity was constitutively activated in germ cell-specific *Nb/Nbl* double mutant mice. This was further supported by a well-documented Notch target gene *Hey1* in germ cells which was markedly elevated (Garcia et al. 2017; Heisig et al. 2012; Wiese et al. 2010). *Hey1* expression is known to be dominantly upregulated in response to sustained Notch activation (Nandagopal et al. 2018). Therefore, the current study confirms previous reports that overaction of the Notch signaling associates with developmental defect in the RT. Moreover, the data reveal a novel finding that dysregulated Notch signaling in germ cells contributes to the malformation of the RT. The results of previous and present studies showed that spermatogenesis was not significantly affected by sustained Notch activation in young adult testes. However, there was a report that overexpression of NICD in murine spermatogonia driven by the *Stra8* (stimulated by retinoic acid 8) promoter increased germ cell apoptosis in aged testes (Huang et al. 2013). This raises the possibility that constitutively Notch activation may have an age-dependent impact on spermatogenesis.

Germ cells are located inside the blood-barrier within seminiferous tubules, raising a question of how aberrant activation of Notch signal in these cells leads to abnormal development of the RT where contains no germ cells. We postulate that certain Notch-regulated factor(s) produce, diffuse and/or secrete from germ cells, transit through the luminal space of the seminiferous tubules to reach the RT where they interact with epithelial cells of the RT to influence the development of the RT. FGF4 appears to be an appropriate candidate. First, FGF4, a soluble/diffusible signaling molecule, is reported to express in germ cells (Yamamoto et al. 2000). It expresses in both prenatal and postnatal testes and increases rapidly after birth. FGF4 has pleiotropic roles in many cell types and maintenance of tissues during embryonic development as well as adult stages (Kosaka et al. 2009). Second, FGF4 is one of several growth factors reported to present in the RT fluid (Hinton et al. 1998; Kirby et al. 2003; Lan et al. 1998). Indeed, it has proposed to be a putative lumicrine factor produced in the seminiferous tubules and acts on downstream reproductive tract (Cotton et al. 2008; Hinton et al. 1998). Third, the expression of *Fgf4* is regulated by Notch. There are several previous reports showing that Notch activity correlated with activation of the FGF signaling pathway and vice versa (Bongarzone et al. 2000; Faux et al. 2001; Katoh and Katoh 2006; Miralles et al. 2006; Small et al. 2003; Wei et al. 2019). Our data demonstrated an inverse relationship between Notch activation and *Fgf4* expression in testicular germ cells. Fourth, blockade of FGFRs induced enlargement of the RT lumens in explant testicular cultures, which was curtailed by additional exogenous FGF4. Lastly and most importantly, germ cell-specific depletion of *Fgf4* recapitulated the phenotype of cystic dilation of the RT, the same as caused by germ cell-specific abolishment of *Nb/Nbl* expression. Overall, the phenotypic similarities of mutants in the Notch and FGF signaling pathways and the complex regulatory relationship suggest that interactions between these two pathways act sequentially in regulation of the RT development. The results of this study do not rule out the possibility that other factors in addition to FGF4 are involved in regulation of the RT development. In fact, at least three FGFs, including FGF2, FGF4 and FGF8 among others that found in the RT fluid (Cotton et al. 2008). A very recent study reported that the RT epithelium expresses FGF9 and FGF10 (Imura-Kishi et al. 2021). These other factors may also be necessary for normal development of the RT. Nevertheless, the present study identifies an association of FGF4/FGFR signaling dysfunction and the cystic dilation of the RT.

Previous studies (Yamamoto et al. 2000) and our data demonstrated that testicular germ cells produce FGF4. However, little is known about the regulation of *Fgf4* expression in germ cells. Our data revealed that the Notch signal activity negatively correlated with *Fgf4* expression in these cells. Depletion of *Nb/Nbl* in germ cells resulted in constitutively activation of Notch and activation of Notch in turn stimulated *Hey1* expression, a best-known primary target of canonical Notch signaling (Garcia et al. 2017; Heisig et al. 2012; Wiese et al. 2010). Hey1 is a basic helix-loop-helix-type transcriptional factor that typically binds to a consensus sequence called E-box (CANNTG) to down-regulate the expression of a number of its target genes (Garcia et al. 2017; Heisig et al. 2012). Interestingly, there are more than ten putative E-boxes found within 2,000 bp of the promoter region upstream from the transcription start site of murine *Fgf4* gene. It is therefore possible that Notch signaling in germ cells down-regulates *Fgf4* expression through the Hey1 transcriptional repressor in the testis.

FGFR3 and FGFR4 are neither essential for prenatal testis development nor crucial for spermatogenesis in the adult (Deng et al. 1996; Weinstein et al. 1998; Yu et al. 2000). *Fgfr1* or *Fgfr2* null mutation resulted in early embryonic lethality (Arman et al. 1998; Deng et al. 1994). Postnatal germ cell-specific double deletion of *Fgfr1* and *Fgfr2*, however, no morphological abnormalities of the testes and functional defects of male fertility were detected in these animals. These findings suggest that FGF4 produced by germ cells may not act as an autocrine/paracrine factor to affect their own functions despite germ cells express *Fgfr1* and *Fgfr2* (Li et al. 2014). It is more likely that FGF4 acts as a lumicrine factor to regulate the development of downstream reproductive tract. This is consistent with the findings that FGF4 mediated by FGFRs and exerts not only autonomous actions but also nonautonomous effects on various tissues (Cotton et al. 2008; Kosaka et al. 2009). It is known that FGF4 binds and activates FGFR1, FGFR2 and FGFR3 at comparable levels (Ornitz et al. 1996). In the explant cultures of neonatal testes, a pan-FGFR inhibitor induced the enlargement of the RT lumen and the luminal enlargement was partially prevented by additional recombinant FGF4, while a selective FGFR4 inhibitor had no effect. These results indicate that FGFRs, possible FGFR1, FGFR2 and FGFR3, mediated FGF4 signaling is indispensable in maintaining normal RT development. However, the identity of the FGFR and the signaling mechanism of FGF4 in the RT remain to be determined.

Morphologically, the RT cells are the simple epithelial cells that comprise the walls of interconnected cavities of the RT. Although the origination of these cells is not unequivocally confirmed (de Mello Santos and Hinton 2019), the results of a few studies suggest that the RT cells are derived from gonadal somatic cells, a common origin of Sertoli cells (Kulibin and Malolina 2020; Malolina and Kulibin 2019; Wrobel 2000). Unlike in the seminiferous tubules of adult testis, the Sertoli cells in the tubuli recti, a short transition zone between the seminiferous tubules and the RT, are capable of proliferation into adulthood (Aiyama et al. 2015; Figueiredo et al. 2016). Our immunohistochemical studies reveal that the RT cells share many markers with Sertoli cells, which include GATA4, SOX9, SF1, WT1 except DMRT1, in addition to their own markers such as PAX8 and CDH1 (Combes et al. 2009; Kulibin and Malolina 2020; Magers et al. 2016; Malolina and Kulibin 2019; Wang et al. 2013). These findings are in line with previous reports that the RT cells are likely differentiated from pre-Sertoli cell lineage (Kulibin and Malolina 2020; Malolina and Kulibin 2019; Wrobel 2000). By adopting and responding to the environment and stimuli, pre-Sertoli cells may transform and epithelize to become RT cells. *Dmrt1* is a conserved autosomal gene expressed by both Sertoli cells and spermatogonia in the developing and adult mammalian testis (Raymond et al. 2000). Analyses of *Dmrt1* null mutant mice unveiled that it may not be crucial in the early stages of gonadogenesis, whereas major defects occurred in testicular maturation after birth in mice (Kim et al. 2007; Raymond et al. 2000). The germ cells failed to enter meiosis and were eventually lost from the epithelium and the pre-Sertoli cells over-proliferated, failed to differentiate to mature Sertoli cells, and eventually died (Raymond et al. 2000). Intriguingly, *Dmrt1* ablation in adult murine testes caused trans-differentiation of matured Sertoli cells into female granulosa-like cells, indicating that DMRT1 is a key factor essential for maintenance of Sertoli cell characteristics in adult testis (Huang et al. 2017; Matson et al. 2011; Matson and Zarkower 2012). Present studies showed that there were only few DMRT1 positive RT cells in the region that were close to the tubuli recti, while the majority of epithelial cells were DMRT1 negative in postnatal RT. Blockade of FGFR induced a significant increase of DMRT1 positive RT cells, which was accompanied by the enlargement of the RT lumen. Similarly, the number of DMRT1 positive RT cells were also elevated in germ cell-specific deleted *Nb/Nbl* and *Fgf4* mice. Based on these findings, we speculate that the disruption of FGF4/FGFR signaling may induce *Dmrt1* expression. Otherwise, *Dmrt1* is normally suppressed or continuously enforced in the RT cells. Aberrant *Dmrt1* expression might reprogram and trans-differentiate the RT cells to more Sertoli-like cells. Consequently, this reverse differentiation or re-programing of RT cells could perturb the development, remodeling and integrity of the RT.

One of the functions of the RT and efferent ductules is reabsorbing luminal fluid. Impairment of fluid reabsorption due to defective development of the efferent ductules causes cystic dilation of the RT. Most notably, inhibition of estrogen signaling or mutation of estrogen receptor ESR1 led to abnormal efferent ductule development. The undifferentiated efferent ductule epithelium exhibits reductions in epithelial height and number of microvilli, loss of lysosomes, and ion exchange and water transport activities (Hess et al. 1997, 2000; Hess and Cooke 2018; Lee et al. 2000). Consequently, backpressure of excessive luminal fluid due to the failure of fluid reabsorption by the efferent ductule epithelium results in cystic dilation of the RT. The cystic dilation of the RT in germ cell-specific *Nb/Nbl* and *Fgf4* mutant mice is unlikely caused by a defective development of the efferent ductules because the morphology of the efferent ductules epithelial cells and ion exchange CFTR and water transports AQP3 and AQP9 in these mutants do not appear to be altered. However, the defective RT development in germ cell-specific *Nb/Nbl* and *Fgf4* mutant mice may cause the aberrant junctional formation of the RT with the seminiferous tubules and/or efferent ductules, which may lead to fluid retention in the RT and give rise to cystic dilation of the lumens. Obviously, further investigation is necessary to elucidate the cellular and molecular mechanisms by which the Notch/FGF4 signaling axis regulates the RT development and the association of the pathogenesis of cystic dilation of the RT.

Cystic dysplasia of the RT is a benign lesion of the testis, presenting primarily in the pediatric population (Friend et al. 2016; Fuchs et al. 2017; Jeyaratnam and Bakalinova 2010; Jones et al. 2000; Leissring and Oppenheimer 1973; Zaragoza et al. 1996). Although it is an uncommon condition in humans, it is also found in a variety of animal species, including cat, dog, fox, horse, rabbit and alpaca (Chambers et al. 2014). Cystic dysplasia of the RT usually manifests itself as a painless scrotal swelling either unilaterally or bilaterally. It is characterized by cystic dilation of the RT whereas spermatogenesis is generally not affected. Common histopathology is multiple, irregular, enlarged cystic spaces lined by the simple cuboidal cells in the testis. However, the etiology of this abnormality is currently unclear (Friend et al. 2016; Fuchs et al. 2017; Gelas et al. 2016; Jeyaratnam and Bakalinova 2010; Leissring and Oppenheimer 1973; Mahlknecht et al. 2015). Notably, the testicular phenotype of *Tex-Cre:Nb*^*f/f*^*/Nbl*^*f/f*^ and *Tex-Cre:Fgf4*^*f/f*^ mice in this study is surprisingly similar to that of cystic dysplasia of the RT in humans. This tempts us to speculate that perturbations of the Notch and/or FGF signaling pathways may associate with the pathogenesis of cystic dysplasia of the RT. Further studies to evaluate the cellular and molecular basis underlying the cystic dilation of the RT in these mouse models may help us to understand the nosogenesis of this rare congenital disorder of the RT in humans and animals.

In summary, the present study uncovers a novel signal pathway in which a cell-autonomous effect of Nb/Nbl on Notch signaling in upstream germ cells couples to regulate a lumicrine factor FGF4 to modulate downstream RT development. Our mouse model demonstrates a functional role of Nb/Nbl in negative modulation of Notch signaling activity in testicular germ cells. Over-activated Notch because of Nb/Nbl deprivation suppresses a testicular germ cell secreted/diffusible factor FGF4. Further studies demonstrated that loss or severe reduction of FGF4 signaling leads to malformation of the RT, providing evidence that FGF4 produced by germ cells, transited through the luminal space to the RT where it mediated by FGFRs, is critical for the differentiation of the RT. The data strongly suggest that proper regulation of Notch signaling by Nb/Nbl in testicular germ cells is important for the maintenance of normal development of the RT.

## Supplementary Information

Below is the link to the electronic supplementary material.

Supplementary file 1 (DOCX 27 KB)

Supplementary file 2 (DOCX 30 KB)

Supplementary file 2 Suppl. Figure 1. Testicular germ cell-specific depletion of Numb/Numbl (Nb/Nbl) in Tex-Cre:Nbf/f/Nblf/f testes. Immunohistochemical staining shows the RT cells are stained positively for GATA4 in both 3 month-old Tex-Cre (A & B) and Tex-Cre:Nbf/f/Nblf/f (C & D) testes. B & E are magnified images of boxed areas in A & C, respectively. Immunohistochemical staining of Nb in adult Tex-Cre testis (E) showing that Nb is localized in all groups of germ cells, Sertoli cells and interstitial cells. In 3 month-old Tex-Cre:Nbf/f/Nblf/f testis, immunostaining of Nb (G & H) and Nbl (I & J) is detected in testicular somatic cells the same as in the adult Tex-Cre testis. However, they are absent in the germ cells. Panel F is a procedure control of immunostaining in which Nb antibody was replaced by a non-specific rabbit IgG. H & J are higher magnified images of G & I. All sections were counterstained with hematoxylin. Suppl. Figure 2. Histological analyses of the testis and efferent ductules of Tex-Cre:Nbf/f/Nblf/f mice. Histological analyses of the testes (A—J) and efferent ductules (K & L) of 3 month-old Tex-Cre (A, B, E, G, I & K) and Tex-Cre:Nbf/f/Nblf/f (C, D, F, H, J & L) mice. H&E (A – D, K & L) staining of testicular (A – D) and efferent ductal sections (K & L) exhibits normal histological appearance in Tex-Cre:Nbf/f/Nblf/f adult mice (C, D & L) compared to Tex-Cre siblings (A, B & K). Immunohistochemical staining of a germ cell marker GCNF (E & F), a Sertoli cell marker GATA4 (G & H) and an interstitial cell marker Cyp17A1 (I & J) does not detect any discernable differences between Tex-Cre:Nbf/f/Nblf/f (E, G & I) and Tex-Cre siblings (F, H & J). B & D and the insets in panels E—L are higher magnified images. Suppl. Figure 3. Fgf4 expression in the mouse testes during postnatal development. RT-PCR results show that Fgf4 mRNAs are readily detectable in the whole testis as well as in purified interstitial, germ and Sertoli cells in adult mice (A). Analyses of Fgf4 expression by RT-PCR during postnatal testicular development reveal that Fgf4 mRNA levels significantly increase during neonatal period and then gradually decrease from pubertal period to adulthood in the testes (B). Western blot results show that FGF4 protein levels are dramatically elevated during neonatal period and remain constant from neonatal period to adulthood in the testes (C) (n = 4). Immunohistochemical staining shows wide distribution of FGF4, including all groups of germ cells and somatic cells (interstitial and Sertoli cells) in the testes from neonatal period to adulthood (D). Control in D is a procedure control of immunostaining in which FGF4 antibody was replaced by a non-specific rabbit IgG. Statistical analysis is performed by One-way ANOVA. *p < 0.05, ***p < 0.001 compared to day-1 (D1) mice. Suppl. Figure 4. GATA4, DMRT1 and PAX8 expression during embryonic to newborn testicular development. H & E staining (A – D) of embryonic (E13.5, E15.5 & E17.5) and day-1 (D1) testes. Nuclear immunostaining of GATA4 (E – H) is observed in Sertoli cells of seminiferous tubules (ST) as well as lining epithelial cells of rete testis (RT). Prominent nuclear immunostaining of DMRT1 (I – L) is primarily found in Sertoli cells of the ST but not in lining epithelial cells of the RT. In contrast, nuclear immunostaining of PAX8 (M – P) is exclusively detected in lining epithelial cells of the RT but not in Sertoli cells of the ST. During embryonic 13.5 to the day of birth, the immunostaining patterns of these proteins in the testes and the RT display no significant changes among all age groups. The insets are magnified images of boxed areas in the corresponding pictures. Suppl. Figure 5. Effects of FGF4 and FGF receptor inhibitor on GATA4, WT1, SF1, AR, PAX8, ESR1, DAX1 and E-Cadherin expression in the RT cells. Immunohistochemical staining results show that treatments of FGF4 (10 – 50 ng) or LY2874455 (0.1 – 1 µM) for 72 h do not alter the immunostaining intensity or localization of GATA4, WT1, SF1, AR, PAX8, ESR1, DAX1 and E-Cadherin in day-1 testicular explants. All sections were counterstained with hematoxylin. The insets are magnified images of boxed areas in the corresponding pictures. ST (Seminiferous tubule), RT (Rete testis). Suppl. Figure 6. Effects of testicular germ cell-specific deletion of Nb/Nbl and Fgf4 on AQP3, AQP9 and CFTR expression in the efferent ductules. Immunohistochemical staining results show similar immunostaining intensity and pattern of AQP3 (A—C), AQP9 (D—F) and CFTR (G—I) in the efferent ductules among 3 month-old Tex-Cre (A, D & E), Tex-Cre:Nbf/f/Nblf/f (B, E & H) and Tex-Cre:Fgf4f/f (C, E & I) mice. All sections were counterstained with hematoxylin. The insets are magnified images of boxed areas in the corresponding pictures

## Abbreviations

RT: Rete testis
Nb: Numb
Nbl: Numb-like
Dll: Delta-like
Jag: Jagged
NICD: Notch intracellular domain
*Hey*: Hes related with YRPW motif
*Hes*: Hairy and enhancer of split
*Lfng*: Lunatic fringe
SF1: Steroidogenic factor 1
AR: Androgen receptor
SOX9: SRY-box transcription factor 9
WT1: Wilms tumor protein
GATA4: GATA binding protein 4
DMRT1: Doublesex and Mab-3 related transcription factor 1
PAX8: Paired box gene-8 protein
CDH1: E-cadherin
FGF: Fibroblast growth factor
FGFR: FGF receptor
Tex-Cre: Tex101-iCre
E: Embryonic day
D: Postnatal day
Sec: Second
min: Minute
hr: Hour
HBSS: Hank's balanced salt solution
DMEM: Dulbecco’s modified eagle medium
FBS: Fetal bovine serum
RT-PCR: Reverse transcription-polymerase chain reaction
GC1: GC1-spg
RIPA: Radioimmunoprecipitation
DAPT: N-[N-(3,5-difluorophenacetyl)-l-alanyl]-S-phenyl glycine t-butyl ester
DMSO: Dimethyl sulfoxide
*Rpl*: Ribosomal protein large subunit
PBS: Phosphate buffered saline
LY: LY2874455
BLU: BLU9931
H&E: Hematoxylin and eosin
GCNF: Germ cell nuclear factor
Cyp17A1: Cytochrome P450 family 17 subfamily A member 1
ESR1: Estrogen receptor 1
MMTV: Mouse mammary tumor virus
*Stra8*: Stimulated by retinoic acid 8
AQP3: Aquaporin 3
AQP9: Aquaporin 9
CFTR: Cystic fibrosis transmembrane conductance regulator
