# Supplementary material for: Dysregulation of Notch-FGF signaling axis in germ cells results in cystic dilation of the rete testis in mice
Source: J Cell Commun Signal. 2021 Jun 8;16(1):75–92. doi: 10.1007/s12079-021-00628-0 (PMC8688682; doi:10.1007/s12079-021-00628-0)
Supplement: Supplementary file 1 — (DOCX 27 KB) [file 12079_2021_628_MOESM1_ESM.docx]

**Suppl. Table 1.** List of PCR primers used for the present study

| **Genes** | **Forward (5’-3’)** | **Reverse (5’-3’)** |
| --- | --- | --- |
| **RT-PCR primers** | | |
| ***Numb*** | TGAACAAACTACGGCAAAGCTTC | TGGGGCACAGAAGGAAACTTTT |
| ***Numbl*** | GGAGACCTGATCAGCATTTGTCC | AGACTTCACGGACTTCCGGC |
| ***Fgf4*** | CTTCGGCGGCTCTACTGCAA | ATACCGGGGTACGCGTAGGATTCG |
| ***Hey1*** | TAGTGAGCTGGACGAGACCATCG | CGTGCGCGTCAAAATAACCT |
| ***Rpl19*** | GAGTATGCTCAGGCTTCAGA | TTCCTTGGTCTTAGACCTGC |
| **Real Time-PCR primers** | | |
| ***Hey1*** | GGTCTGTTTCCTGGTGTGTATAA | CACCTCGGTCCATCAAAGTAG |
| ***Fgf4*** | CTTTGGAATAGCGGAACAGA | CGACAGGGTTCATACCAAAG |
| ***Rpl13*** | ATGACAAGAAAAAGCGGATG | CTTTTCTGCCTGTTTCCGTA |
| **Genotyping PCR primers** | | |
| ***Tex-Cre*** | TCTGATGAAGTCAGGAAGAACC | GAGATGTCCTTCACTCTGATTC |
| ***Numb*** | GAAGGAGCCTTCCAAAATCGTATTC | AGGCTTCTGGGAAACCTCACTTACT |
| ***Numbl*** | CTGTTTCCTGCCTTCCTTCCTAGTC | GCAGGCAGTGAAAAACCATCTCTC |
| ***Fgf4*** | TCTGGAGAGGAACTAGGAATGG | GAAGAGAAGCAGGCAGATGC |

**Suppl. Table 2.** List of antibodies used in the present study

| **Antibody** | **Host** | **Dilution** | **Manufacturer** |
| --- | --- | --- | --- |
| Activated Notch1 | Rabbit | 1:150 | Abcam |
| Hey1 | Rabbit | 1:2,000 | Proteintech |
| FGF4 | Rabbit | 1:3,000 | Boster BioTech |
| Numb | Rabbit | 1:800 | Proteintech |
| Numb-like | Rabbit | 1:2,000 | Proteintech |
| DMRT1 | Mouse | 1:100 | Santa Cruz Biotech |
| SF1 | Mouse | 1:100 | Santa Cruz Biotech |
| ESR1 | Rabbit | 1:100 | Santa Cruz Biotech |
| WT1 | Rabbit | 1:100 | Santa Cruz Biotech |
| AR | Rabbit | 1:100 | Santa Cruz Biotech |
| GATA4 | Rabbit | 1:100 | Santa Cruz Biotech |
| Cyp17A1 | Rabbit | 1:100 | Santa Cruz Biotech |
| GCNF | Rabbit | 1:100 | Santa Cruz Biotech |
| DAX1 | Rabbit | 1:100 | USB Biological |
| PAX8 | Rabbit | 1:800 | Proteintech |
| E-Cadherin | Rabbit | 1:1,000 | Cell Signaling |
| AQP3 | Mouse | 1:100 | Santa Cruz Biotech |
| AQP9 | Mouse | 1:100 | Santa Cruz Biotech |
| CFTR | Mouse | 1:100 | Santa Cruz Biotech |
| β-Tubulin | Mouse | 1:3,000 | Sigma-Millipore |
| β-Actin | Mouse | 1:3,000 | Santa Cruz Biotech |
