# Supplementary material for: Dysregulation of Notch-FGF signaling axis in germ cells results in cystic dilation of the rete testis in mice
Source: J Cell Commun Signal. 2021 Jun 8;16(1):75–92. doi: 10.1007/s12079-021-00628-0 (PMC8688682; doi:10.1007/s12079-021-00628-0)
Supplement: Supplementary file 2 — (DOCX 30 KB) [file 12079_2021_628_MOESM2_ESM.docx]

**Supplemental figure legends**

**Suppl. Fig. 1. Testicular germ cell-specific depletion of Numb/Numbl (Nb/Nbl) in *Tex-Cre:Nb^f/f^/Nbl^f/f^* testes.** Immunohistochemical staining shows the RT cells are stained positively for GATA4 in both 3 month-old *Tex-Cre* (A & B) and *Tex-Cre:Nb^f/f^/Nbl^f/f^* (C & D) testes. B & E are magnified images of boxed areas in A & C, respectively. Immunohistochemical staining of Nb in adult *Tex-Cre* testis (E) showing that Nb is localized in all groups of germ cells, Sertoli cells and interstitial cells. In 3 month-old *Tex-Cre:Nb^f/f^/Nbl^f/f^* testis, immunostaining of Nb (G & H) and Nbl (I & J) is detected in testicular somatic cells the same as in the adult *Tex-Cre* testis. However, they are absent in the germ cells. Panel F is a procedure control of immunostaining in which Nb antibody was replaced by a non-specific rabbit IgG. H & J are higher magnified images of G & I. All sections were counterstained with hematoxylin.

**Suppl. Fig. 2. Histological analyses of the testis and efferent ductules of *Tex-Cre:Nb^f/f^/Nbl^f/f^* mice.** Histological analyses of the testes (A - J) and efferent ductules (K & L) of 3 month-old *Tex-Cre* (A, B, E, G, I & K) and *Tex-Cre:Nb^f/f^/Nbl^f/f^* (C, D, F, H, J & L) mice. H & E (A – D, K & L) staining of testicular (A – D) and efferent ductal sections (K & L) exhibits normal histological appearance in *Tex-Cre:Nb^f/f^/Nbl^f/f^* adult mice (C, D & L) compared to *Tex-Cre* siblings (A, B & K). Immunohistochemical staining of a germ cell marker GCNF (E & F), a Sertoli cell marker GATA4 (G & H) and an interstitial cell marker Cyp17A1 (I & J) does not detect any discernable differences between *Tex-Cre:Nb^f/f^/Nbl^f/f^* (E, G & I) and *Tex-Cre* siblings (F, H & J). B & D and the insets in panels E - L are higher magnified images.

**Suppl. Fig. 3. *Fgf4* expression in the mouse testes during postnatal development.** RT-PCR results show that *Fgf4* mRNAs are readily detectable in the whole testis as well as in purified interstitial, germ and Sertoli cells in adult mice (A). Analyses of *Fgf4* expression by RT-PCR during postnatal testicular development reveal that *Fgf4* mRNA levels significantly increase during neonatal period and then gradually decrease from pubertal period to adulthood in the testes (B). Western blot results show that FGF4 protein levels are dramatically elevated during neonatal period and remain constant from neonatal period to adulthood in the testes (C) (n = 4). Immunohistochemical staining shows wide distribution of FGF4, including all groups of germ cells and somatic cells (interstitial and Sertoli cells) in the testes from neonatal period to adulthood (D). Control in D is a procedure control of immunostaining in which FGF4 antibody was replaced by a non-specific rabbit IgG. Statistical analysis is performed by One-way ANOVA. *p<0.05, ***p<0.001 compared to day-1 (D1) mice.

**Suppl. Fig. 4. GATA4, DMRT1 and PAX8 expression during embryonic to newborn testicular development.** H & E staining (A – D) of embryonic (E13.5, E15.5 & E17.5) and day-1 (D1) testes. Nuclear immunostaining of GATA4 (E – H) is observed in Sertoli cells of seminiferous tubules (ST) as well as lining epithelial cells of rete testis (RT). Prominent nuclear immunostaining of DMRT1 (I – L) is primarily found in Sertoli cells of the ST but not in lining epithelial cells of the RT. In contrast, nuclear immunostaining of PAX8 (M – P) is exclusively detected in lining epithelial cells of the RT but not in Sertoli cells of the ST. During embryonic 13.5 to the day of birth, the immunostaining patterns of these proteins in the testes and the RT display no significant changes among all age groups. The insets are magnified images of boxed areas in the corresponding pictures.

**Suppl. Fig. 5. Effects of FGF4 and FGF receptor inhibitor on GATA4, WT1, SF1, AR, PAX8, ESR1, DAX1 and E-Cadherin expression in the RT cells**. Immunohistochemical staining results show that treatments of FGF4 (10 – 50 ng) or LY2874455 (0.1 – 1 µM) for 72 hrs do not alter the immunostaining intensity or localization of GATA4, WT1, SF1, AR, PAX8, ESR1, DAX1 and E-Cadherin in day-1 testicular explants. All sections were counterstained with hematoxylin. The insets are magnified images of boxed areas in the corresponding pictures. ST (Seminiferous tubule), RT (Rete testis).

**Suppl. Fig. 6. Effects of testicular germ cell-specific deletion of *Nb/Nbl* and *Fgf4* on AQP3, AQP9 and CFTR expression in the efferent ductules.** Immunohistochemical staining results show similar immunostaining intensity and pattern of AQP3 (A - C), AQP9 (D - F) and CFTR (G - I) in the efferent ductules among 3 month-old *Tex-Cre* (A, D & E), *Tex-Cre:Nb^f/f^/Nbl^f/f^* (B, E & H) and *Tex-Cre:Fgf4^f/f^* (C, E & I) mice. All sections were counterstained with hematoxylin. The insets are magnified images of boxed areas in the corresponding pictures.
