# Supplementary material for: Dysregulation of Notch-FGF signaling axis in germ cells results in cystic dilation of the rete testis in mice
Source: J Cell Commun Signal. 2021 Jun 8;16(1):75–92. doi: 10.1007/s12079-021-00628-0 (PMC8688682; doi:10.1007/s12079-021-00628-0)
Supplement: Supplementary file 3 — Supplementary file 2 Suppl. Figure 1. Testicular germ cell-specific depletion of Numb/Numbl (Nb/Nbl) in Tex-Cre:Nbf/f/Nblf/f testes. Immunohistochemical staining shows the RT cells are stained positively for GATA4 in both 3 month-old Tex-Cre (A & B) and Tex-Cre:Nbf/f/Nblf/f (C & D) testes. B & E are magnified images of boxed areas in A & C, respectively. Immunohistochemical staining of Nb in adult Tex-Cre testis (E) showing that Nb is localized in all groups of germ cells, Sertoli cells and interstitial cells. In 3 month-old Tex-Cre:Nbf/f/Nblf/f testis, immunostaining of Nb (G & H) and Nbl (I & J) is detected in testicular somatic cells the same as in the adult Tex-Cre testis. However, they are absent in the germ cells. Panel F is a procedure control of immunostaining in which Nb antibody was replaced by a non-specific rabbit IgG. H & J are higher magnified images of G & I. All sections were counterstained with hematoxylin. Suppl. Figure 2. Histological analyses of the testis and efferent ductules of Tex-Cre:Nbf/f/Nblf/f mice. Histological analyses of the testes (A—J) and efferent ductules (K & L) of 3 month-old Tex-Cre (A, B, E, G, I & K) and Tex-Cre:Nbf/f/Nblf/f (C, D, F, H, J & L) mice. H&E (A – D, K & L) staining of testicular (A – D) and efferent ductal sections (K & L) exhibits normal histological appearance in Tex-Cre:Nbf/f/Nblf/f adult mice (C, D & L) compared to Tex-Cre siblings (A, B & K). Immunohistochemical staining of a germ cell marker GCNF (E & F), a Sertoli cell marker GATA4 (G & H) and an interstitial cell marker Cyp17A1 (I & J) does not detect any discernable differences between Tex-Cre:Nbf/f/Nblf/f (E, G & I) and Tex-Cre siblings (F, H & J). B & D and the insets in panels E—L are higher magnified images. Suppl. Figure 3. Fgf4 expression in the mouse testes during postnatal development. RT-PCR results show that Fgf4 mRNAs are readily detectable in the whole testis as well as in purified interstitial, germ and Sertoli cells in adult mice (A). Analys [file 12079_2021_628_MOESM3_ESM.pptx]

## Slide 1
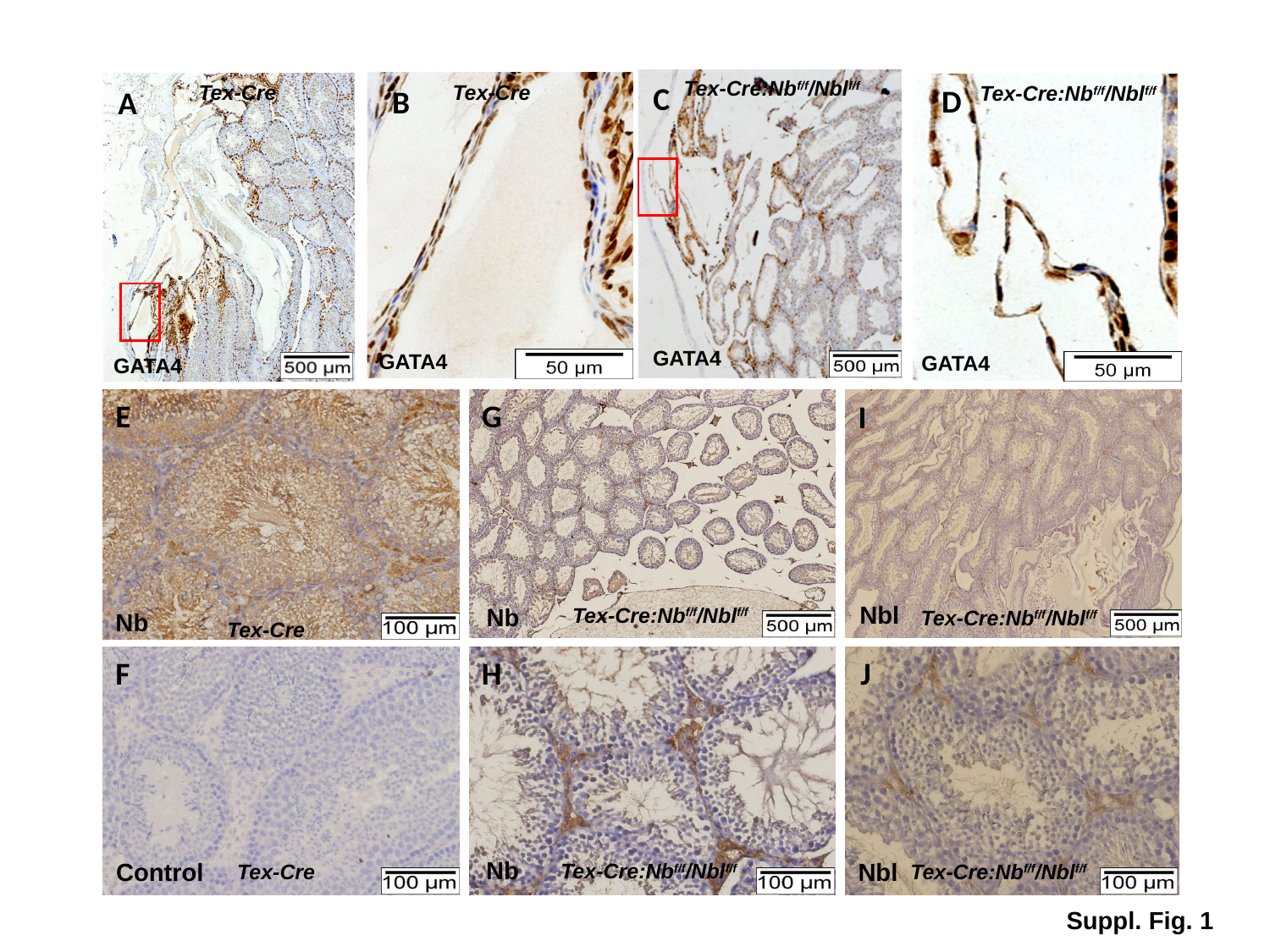

Tex-Cre:Nbf/f/Nblf/f
C
Tex-Cre
Tex-Cre
Tex-Cre:Nbf/f/Nblf/f
D
B
A
GATA4
GATA4
GATA4
GATA4
E
G
I
Nbl
Nb
Nb
F
H
J
Nb
Control
Nbl
Tex-Cre:Nbf/f/Nblf/f
Tex-Cre:Nbf/f/Nblf/f
Tex-Cre
Tex-Cre:Nbf/f/Nblf/f
Tex-Cre:Nbf/f/Nblf/f
Tex-Cre
Suppl. Fig. 1

## Slide 2
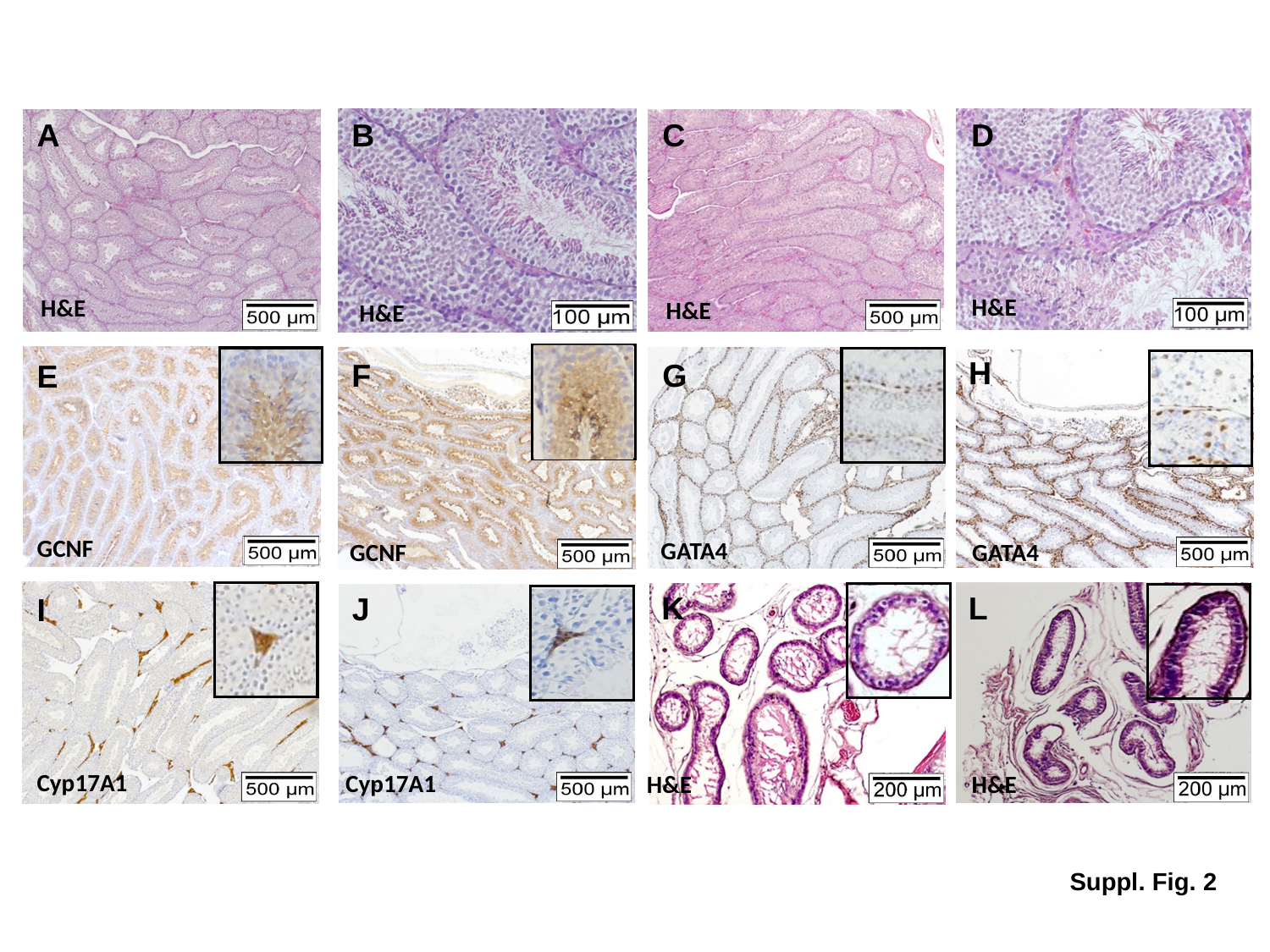

C
B
D
A
H&E
H&E
H&E
H&E
H
E
G
F
GCNF
GATA4
GATA4
GCNF
K
L
I
J
Cyp17A1
Cyp17A1
H&E
H&E
Suppl. Fig. 2

## Slide 3
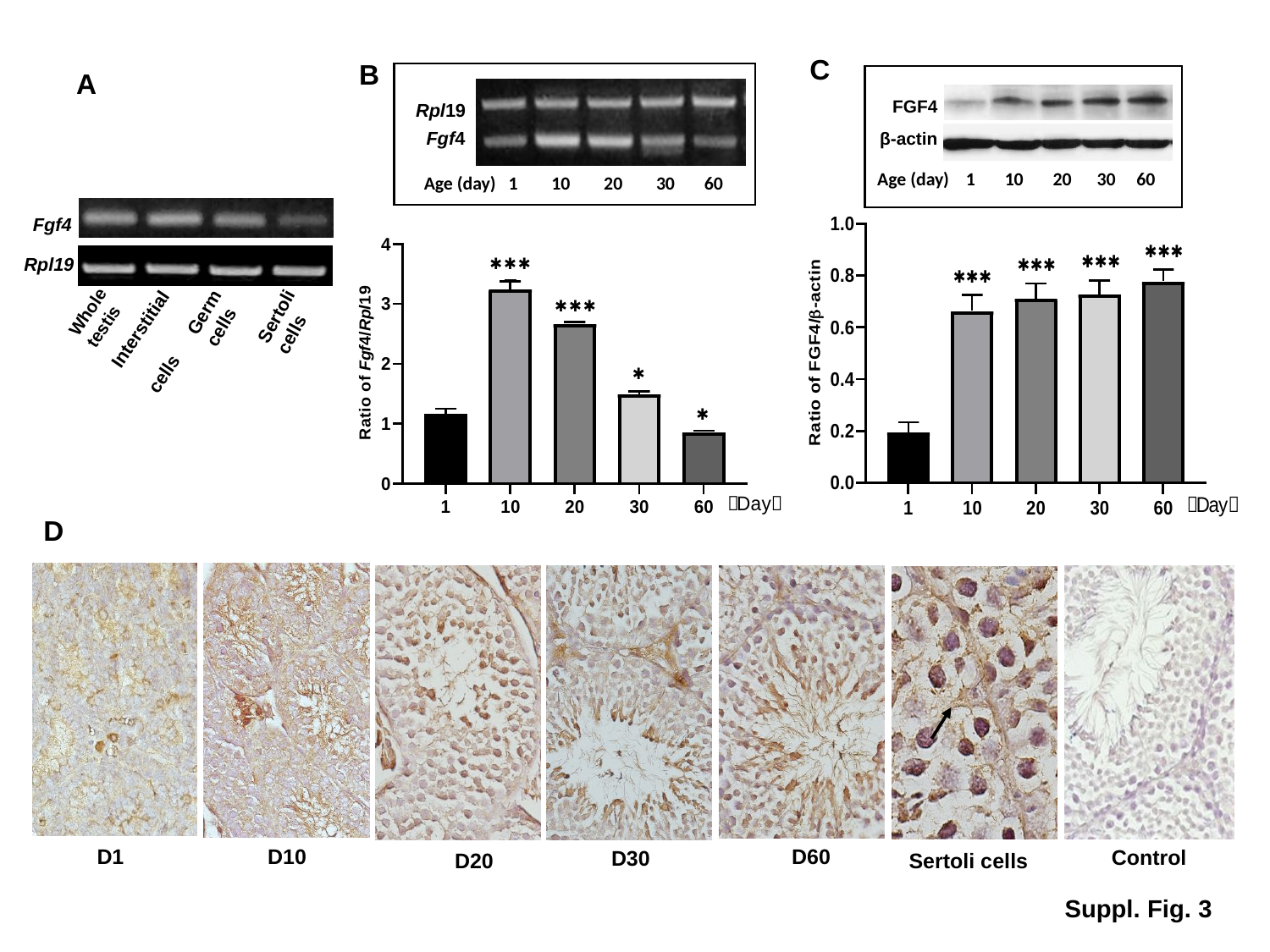

C
B
A
Rpl19
Fgf4
Age (day) 1 10 20 30 60
FGF4
β-actin
Age (day) 1 10 20 30 60
Fgf4
Germ cells
Whole testis
Sertoli cells
Interstitial cells
Rpl19
D
D10
D1
D60
Control
D30
D20
Sertoli cells
Suppl. Fig. 3

## Slide 4
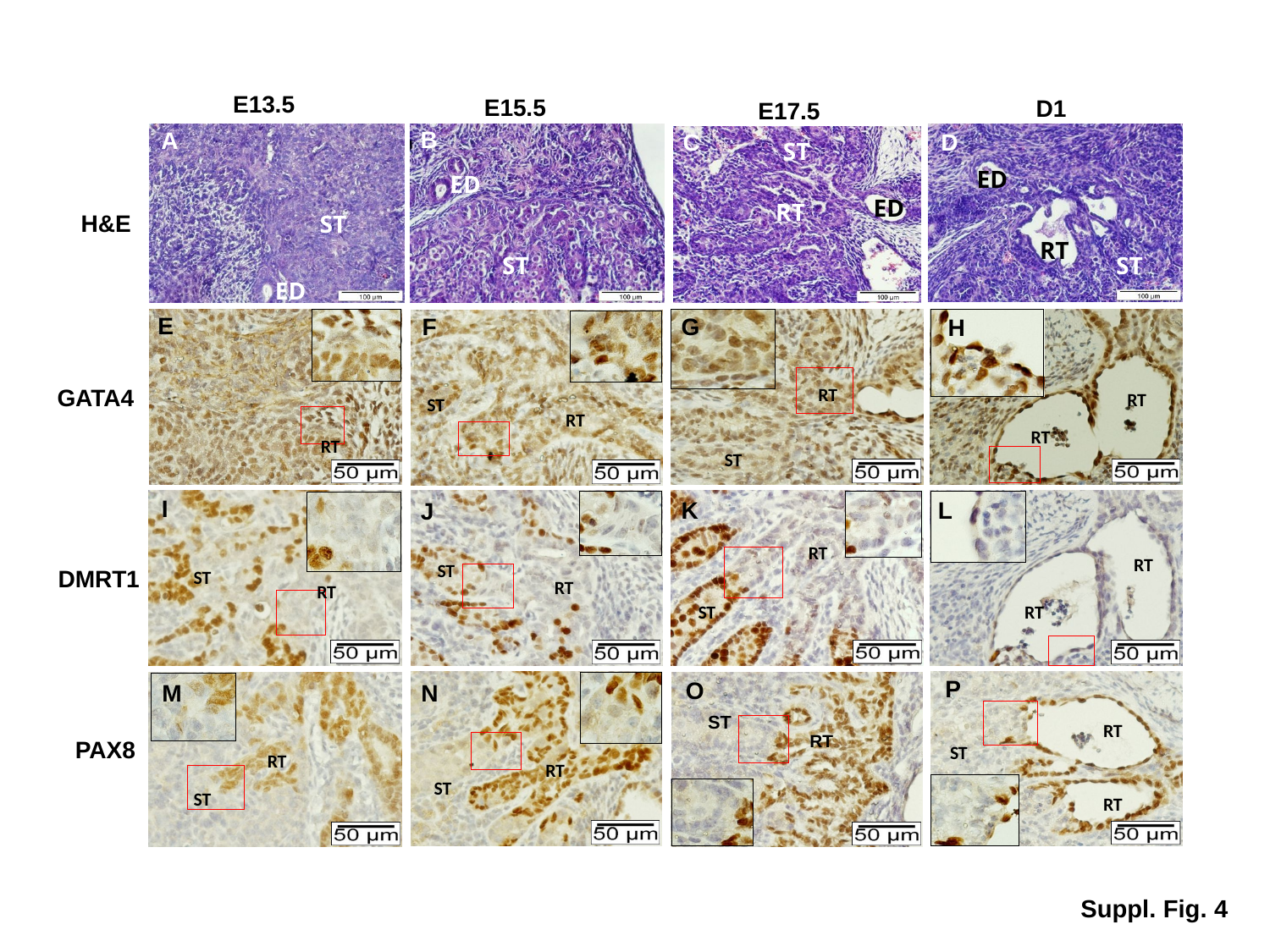

E13.5
E15.5
D1
E17.5
E
RT
G
RT
ST
F
ST
RT
H
RT
RT
I
ST
RT
L
RT
RT
K
RT
ST
J
ST
RT
P
RT
ST
RT
O
ST
RT
M
RT
ST
N
RT
ST
B
A
D
C
ST
ED
ED
ED
RT
ST
RT
ST
ST
ED
H&E
GATA4
DMRT1
PAX8
Suppl. Fig. 4

## Slide 5
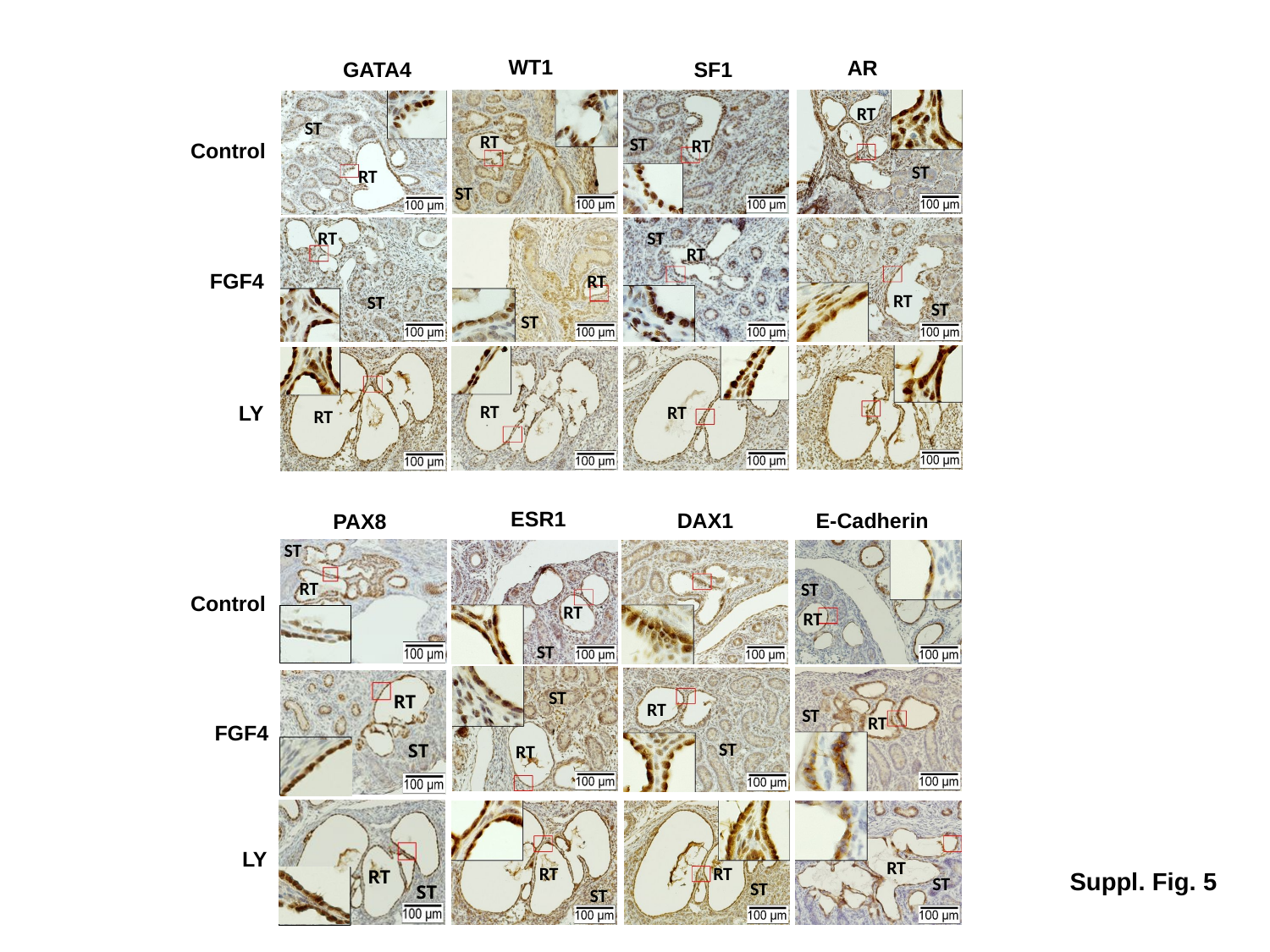

WT1
AR
SF1
GATA4
RT
ST
RT
ST
RT
Control
ST
RT
ST
RT
ST
RT
FGF4
RT
RT
ST
ST
ST
LY
RT
RT
RT
RT
ESR1
E-Cadherin
DAX1
PAX8
ST
RT
RT
ST
Control
RT
RT
ST
ST
ST
RT
ST
RT
 FGF4
ST
RT
LY
RT
RT
RT
ST
ST
ST
RT
ST
RT
Suppl. Fig. 5
ST

## Slide 6
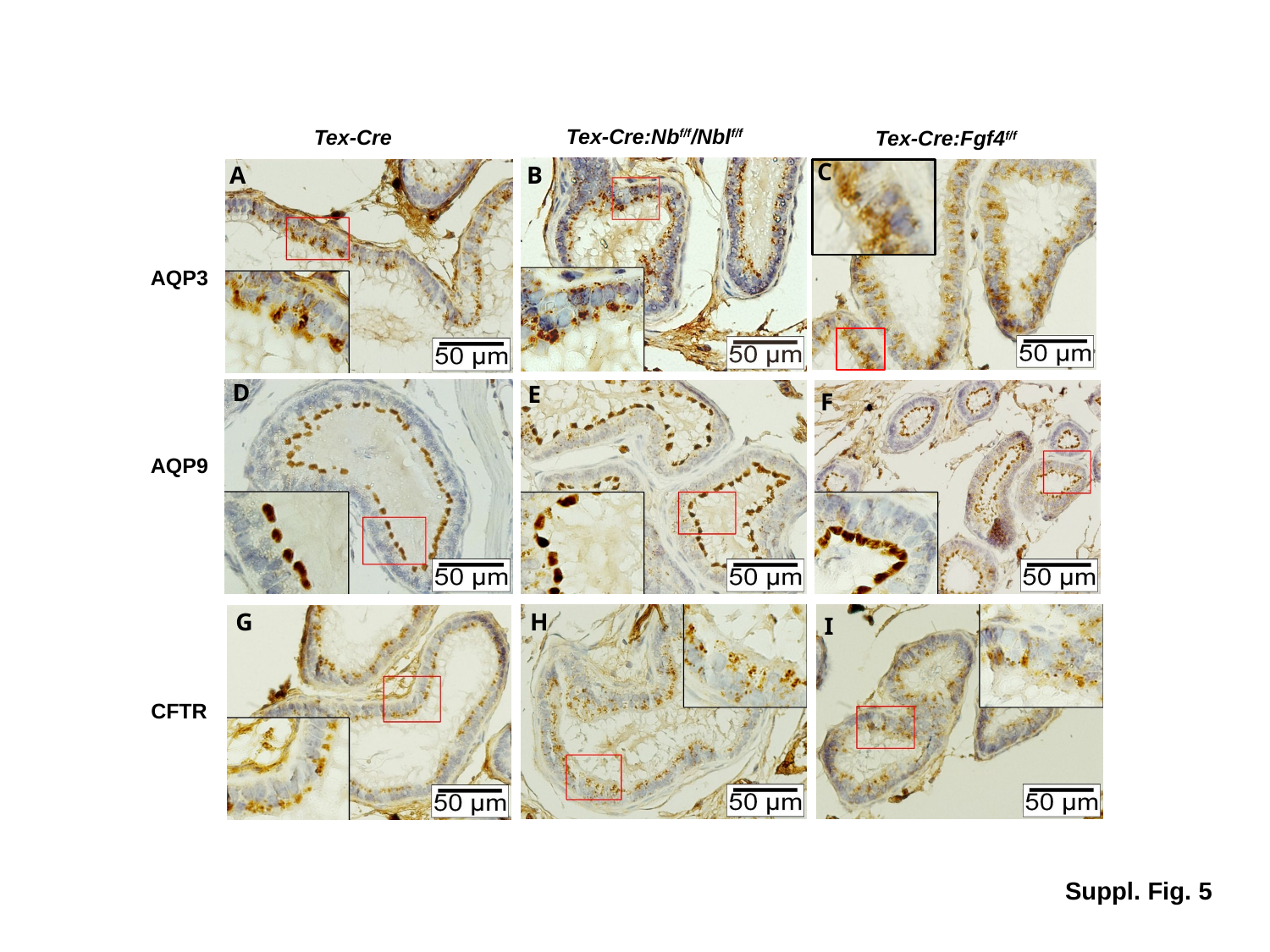

Tex-Cre:Nbf/f/Nblf/f
Tex-Cre
Tex-Cre:Fgf4f/f
C
B
A
AQP3
D
E
F
AQP9
H
G
I
CFTR
Suppl. Fig. 5
